# The psychedelic afterglow phenomenon: a systematic review of subacute effects of classic serotonergic psychedelics

**DOI:** 10.1177/20451253231172254

**Published:** 2023-05-29

**Authors:** Ricarda Evens, Marianna Elisa Schmidt, Tomislav Majić, Timo Torsten Schmidt

**Affiliations:** Department of Psychology, Humboldt-Universität zu Berlin, Berlin, Germany; Department of Psychiatry and Neurosciences, Charité – Universitätsmedizin Berlin, Corporate Member of Freie Universität Berlin and Humboldt-Universität zu Berlin, Berlin, Germany; Psychedelic Substances Research Group, Psychiatric University Clinic of Charité at St. Hedwig Hospital, Charité – Universitätsmedizin Berlin, Unter den Linden 6, Berlin 10099, Germany; Department of Education and Psychology, Freie Universität Berlin, Berlin, Germany; Department of Psychiatry and Neurosciences, Charité – Universitätsmedizin Berlin, Corporate Member of Freie Universität Berlin and Humboldt-Universität zu Berlin, Berlin, Germany; Psychedelic Substances Research Group, Psychiatric University Clinic of Charité at St. Hedwig Hospital, Charité – Universitätsmedizin Berlin, Berlin, Germany; Psychedelic Substances Research Group, Psychiatric University Clinic of Charité at St. Hedwig Hospital, Charité – Universitätsmedizin Berlin, Berlin, Germany; Department of Education and Psychology, Freie Universität Berlin, Berlin, Germany

**Keywords:** afterglow, hallucinogen, psychedelics, serotonergic, subacute

## Abstract

**Background:**

Classic serotonergic psychedelics have anecdotally been reported to show a characteristic pattern of subacute effects that persist after the acute effects of the substance have subsided. These transient effects, sometimes labeled as the ‘psychedelic afterglow’, have been suggested to be associated with enhanced effectiveness of psychotherapeutic interventions in the subacute period.

**Objectives:**

This systematic review provides an overview of subacute effects of psychedelics.

**Methods:**

Electronic databases (MEDLINE, Web of Science Core Collection) were searched for studies that assessed the effects of psychedelics (LSD, psilocybin, DMT, 5-MeO-DMT, mescaline, or ayahuasca) on psychological outcome measures and subacute adverse effects in human adults between 1950 and August 2021, occurring between 1 day and 1 month after drug use.

**Results:**

Forty-eight studies including a total number of 1,774 participants were eligible for review. Taken together, the following subacute effects were observed: reductions in different psychopathological symptoms; increases in wellbeing, mood, mindfulness, social measures, spirituality, and positive behavioral changes; mixed changes in personality/values/attitudes, and creativity/flexibility. Subacute adverse effects comprised a wide range of complaints, including headaches, sleep disturbances, and individual cases of increased psychological distress.

**Discussion:**

Results support narrative reports of a subacute psychedelic ‘afterglow’ phenomenon comprising potentially beneficial changes in the perception of self, others, and the environment. Subacute adverse events were mild to severe, and no serious adverse events were reported. Many studies, however, lacked a standardized assessment of adverse effects. Future studies are needed to investigate the role of possible moderator variables and to reveal if and how positive effects from the subacute window may consolidate into long-term mental health benefits.

## Introduction

A substantial number of clinical trials are currently conducted to explore the safety and efficacy of classic serotonergic hallucinogens [‘psychedelics’, for example, lysergic acid diethylamide (LSD), psilocybin, or ayahuasca] as therapeutic agents. Previous findings are encouraging, with preliminary evidence for positive effects of psychedelics on major depression, existential distress in life-threatening illnesses, and substance-use disorders.^1–6^ In contrast to most other substances known in traditional psychopharmacology, psychedelics show a unique pattern of postacute effects that persist or occur after acute effects have subsided, and therapeutic effects have been observed already after one or few treatment sessions. Those postacute effects have been described to follow a specific temporal progression that can be divided into subacute and long-term effects.^7^ Effects in the subacute window have sometimes been referred to as ‘psychedelic afterglow’, a term coined in the 1960s.^8^

Pahnke and colleagues described that in this period ‘mood is elevated and energetic; there is a relative freedom from concerns of the past and from guilt and anxiety, and the disposition and capacity to enter into close interpersonal relationships is enhanced’ (p. 1858).^9^ Other reports depict this period as a radiant and positive feeling of well-being that often connotes a real change in values, an increase in spirituality, a decrease in meaningless goals, less emphasis on material things, a feeling of being more at home in life and a greater appreciation of life’s possibilities. (p. 283)^10^ or as ‘a carry-over period marked by increased openness and willingness to communicate’ (p. 1249)^11^ and that offers ‘a unique opportunity for effective psychotherapeutic work’ (p. 1858).^9^ The ‘psychedelic afterglow’ has been described to be transient and to variably ‘persist from two weeks to a month and gradually fade into vivid memories’ (p. 1858).^9^ Those subacute effects may then completely subside or transition into long-term or residual effects that have been observed to last months or possibly even years.^12^

Although the psychedelic afterglow phenomenon is anecdotally well known, its descriptions are primarily based on individual case reports. To our knowledge, and in contrast to acute^13,14^ and long-term effects of psychedelics,^15^ there is no systematic review available that selectively and comprehensively summarizes subacute effects of psychedelics. One recent meta-analysis focused on postacute psychological effects, reporting large effect sizes on a range of outcomes, including targeted symptoms within psychiatric samples, negative and positive affect-related measures, social outcomes, and existential/spiritual outcomes.^16^ However, this work combined subacute with long-term effects, preventing conclusions restricted to effects specific to the subacute period. As previous reports on the subacute psychedelic afterglow emphasize its transient nature, subacute and long-term effects may differ in quantity and/or quality. Therefore, we aimed to provide an overview specifically focused on psychological phenomena observed in the subacute period after psychedelic substance use.

Two symptom-specific reviews reported subacute effects separately from long-term effects and observed rapid antidepressant^17,18^ and anxiolytic effects^17^ after psychedelic use that were largely sustained also in more extended follow-up periods. Based on the previous descriptions of the psychedelic afterglow, we hypothesized, however, that there might be an even broader spectrum of subacute effects. Using the method of an exploratory, systematic review, we collected all reports on subacute effects after psychedelic use in the domain of psychological outcome measures. Specifically, we wanted to explore whether findings coincide with the largely positive narrative descriptions of the psychedelic afterglow or whether adverse effects have been observed, too. The review process considered all human studies in adult populations published between 1950 and August 2021. As the focus was to get an exhaustive overview of all possible subacute effects of psychedelics that have been previously reported, we did not apply restrictions toward the study population or study type, as long as subacute effects were presented in comparison to baseline data. Since a thorough description of potential harms is essential for a comprehensive evaluation of possible clinical benefits of psychedelics, and even rarely or sporadically occurring harms could limit its clinical use, we additionally collected all reports of subacute adverse effects of psychedelics, irrespective of the form of collection and whether they occurred on individual or group level.

## Methods

The present study followed the guidelines for systematic reviews and meta-analyses described in the PRISMA Statement.^19^

### Eligibility criteria

The review included studies that assessed the effects of psychedelics on psychological outcome measures in a subacute follow-up period in human adult samples, including clinical and nonclinical populations. Subacute effects are usually described to last from days up to a few weeks.^9,12,20^ To capture all subacute effects, the subacute follow-up in this review was defined as a period between 1 day and 1 month after drug ingestion. The following psychedelics were included: lysergic acid diethylamide (LSD), psilocybin, N, N-dimethyltryptamine (DMT), 5-methoxy-dimethyltryptamine (5-MeO-DMT), mescaline, and ayahuasca (a plant concoction containing a combination of DMT and monoamine oxidase inhibitors). To ensure that the reported effects were relatable to psychedelic substances, studies were excluded if the study protocol requested to terminate the acute psychedelic experience for all participants artificially by default using antipsychotic drugs (e.g. chlorpromazine hydrochloride) or if the study assessed micro-dosing (i.e. the use of very low doses that do not produce clearly noticeable psychedelic experiences). No restrictions on study type were imposed, and the review included data from observational and laboratory studies. However, study designs must have entailed the assessment of baseline data before drug administration, and a pre–post analysis must have been provided. Psychological outcomes were limited to data collected with standardized and published assessment tools. Projective test outcomes were excluded. All reports of subacute adverse effects were included, irrespective of the form of collection.

The review considered articles that provide original data published between 1950 and August 2021. The language was restricted to English and German. Since the review processes of older journals cannot always be retraced, peer review was not an explicit inclusion criterion. However, research databases used for literature searches (see below) almost exclusively list peer-reviewed articles. Book chapters, poster abstracts, case reports, reviews, and comments were excluded. If original data were published more than once (e.g. with different sample sizes of an ongoing trial), data were included only once in the data summary. Studies were excluded if the follow-up assessment point was not clearly defined ( ‘several weeks after the sessions’).

### Information sources and search

Studies were identified by searching the electronic databases MEDLINE *via* PubMed, Web of Science Core Collection *via* Web of Science, and reference lists of selected articles. The last search was performed on August 17, 2021. See supplementary material for the precise search terms used.

### Study selection

The process for selecting studies included the following steps: (1) combination of search results from the two databases, (2) removal of duplicates, (3) screening of titles and abstracts, and (4) assessment of full-text articles to check for study eligibility. Study selection was performed by the author MES and double-checked by RE. In case of disagreement on study eligibility, discrepancies were resolved by consensus.

### Data collection and extracted variables

A data extraction sheet was developed and refined after pilot testing with five studies. Data extraction was performed by MES and RE half each, and results were cross-checked by the other extractor. Disagreements were resolved by consensus. Data extraction was restricted to information provided in the published articles. The following study-level variables were extracted: author, year, study type, psychedelic substance, dosage, population, sample size, age, sex, existence of control group, subacute follow-up latency, psychological domain investigated, name of specific outcome measure, and outcome.

### Risk of bias

Since this review aimed to gain a comprehensive picture of the whole range of subacute effects reported previously in scientific studies, all study types with different levels of internal validity were included. This might result in an increased heterogeneity in study results. To allow the exploration of this heterogeneity, the study type was classified into one of three categories (see legend of Table 2). On the outcome level, the risk of bias was reduced by including only data collected using standardized and validated assessment tools. Adverse effects, on the other hand, were collected very sensitively as even rare and sporadic occurring harms have high clinical significance. Furthermore, only effects that reached statistical significance (i.e. no trends) were summarized.

### Summary measures and synthesis of results

For each study and each psychological outcome, results were grouped into one of four categories: ‘no change’, ‘increase’, ‘decrease’, and ‘other change’. This rating was based on the report of group-level differences between baseline and subacute assessment points, reported as statistically significant in the respective publication. In studies with control groups, this could also have included significant interactions between drug and assessment points. On the review level, data were then summarized according to the psychological domain investigated (e.g. depression, anxiety). To explore the progression of subacute effects, a second summary of results was performed, separately for three different subacute follow-up periods (1 to 2 days, 3 to 14 days, and 15 days to 1 month). Furthermore, differences between clinical and nonclinical populations, and between different classic psychedelic compounds were explored.

## Results

### Study selection and sample characteristics

**Figure 1.**
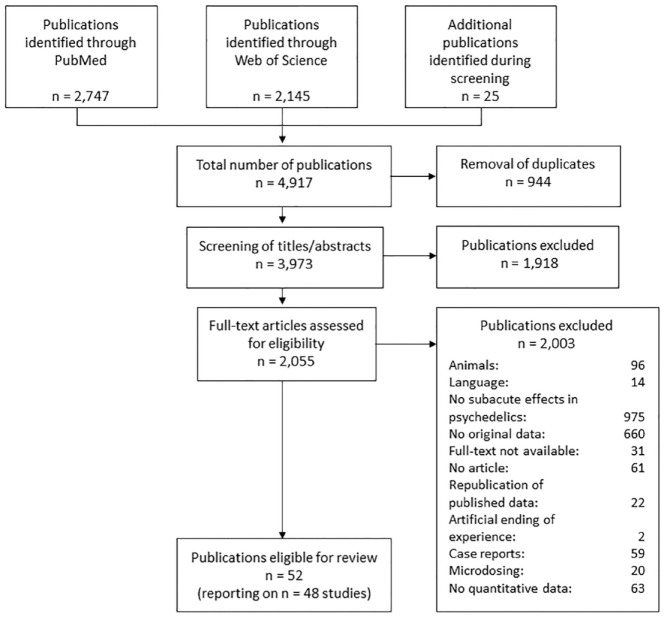
Flow chart of the study selection process.

After removing duplicates, the literature search generated a total number of 3973 publications that were screened for study eligibility. Of those, 1918 publications were excluded after the screening of title and abstract; another 2003 publications were excluded after full-text screening, yielding a total number of 52 publications reporting on 48 studies eligible for data extraction. Further information on the study selection process is provided as a flow chart in Figure 1. The study characteristics of each individual study are displayed in Table 1. Seven articles were published between 1958 and 1971, followed by a period of no eligible publications between 1972 and 1998. Furthermore, 45 articles were published between 1999 and August 2021. Of the 48 studies, 16 investigated psilocybin, 16 ayahuasca, 10 LSD, two 5-MeO-DMT, three different psychedelics, and one mescaline sulfate. Most of the early studies (86%) administered LSD, and most of the modern studies investigated psilocybin (39%) or ayahuasca (39%). All studies combined compose data from 1774 participants. The sample size ranged from 6 to 315, with a mean sample size of 38 (median = 18). Seventeen studies (35%) assessed clinical samples, and 31 studies (65%) assessed nonclinical samples (healthy controls or unselected convenience samples). The subacute follow-up period ranged from 1 day to 1 month; 83% of the studies had at least one subacute assessment point shorter than 2 weeks.

**Table 1.** Sample characteristics of included studies.

Author	Year	Drug	Dosage (ROA)	Population	Sample size (N)	Control	Subacute follow-up	Outcome measures
Denber and West^21^	1958	Mescaline	0.5 g (injection)	Psychiatric patients	9	No	1 day	Personality
Lebovits *et al.*^22^	1960	LSD	100 µg (oral)	Nonclinical	10	Yes	3–9 days	Mood
Ramsay *et al*.^23^	1963	LSD	n/a	Alcohol addiction	47	No	1–2 days	Values
McGlothlin *et al*.^24^	1964	LSD	200 µg (oral)	Nonclinical	15	Yes	1 week	Creativity, social desirability
Bottrill^25^	1969	LSD	400 µg (oral)	Nonclinical	8	Yes	1 week	Personality
Ludwig *et al*.^26^	1969	LSD	3 µg/kg (oral)	Alcohol addiction	132^a^	Yes	10 days– 4 weeks	Personality
Kurland *et al*.^27^	1971	LSD	450 µg (oral)	Alcohol addiction	90	Yes	1 week	Personality, cognitive performance
Gouzoulis-Mayfrank *et al*.^28^	1999	Psilocybin	0.2 mg/kg < 15 mg total (oral)	Nonclinical	8	Yes	1–7 days	Depression, mania, anxiety, Psychosis, complaints
Hasler *et al.*^29^	2002	Psilocybin	212 ± 25 µg/kg (oral)	Nonclinical	8	No	1 week	Complaints
Hasler *et al*.^30^	2004	Psilocybin	45, 115, 215, 315 µg/kg (oral)	Nonclinical	8	Yes	1 day after each session	Mood
Barbosa *et al*.^31^	2005	Ayahuasca	n/a (oral)	Nonclinical	28	No	7–14 days	General psychopathology
Moreno *et al*.^32^	2006	Psilocybin	25, 100, 200, 300 µg/kg (oral)	OCD	9	No	1 day after each session	Obsessive-compulsive symptoms
Trichter *et al*.^33^	2009	Ayahuasca	n/a	Nonclinical	49	Yes	1–4 weeks	Wellbeing, mysticism
Griffiths *et al.*^34^	2011	Psilocybin	5, 10, 20, 30 mg/70 kg (oral)	Nonclinical	18	Yes	3–4 weeks after each session	Mood, attitudes, social effects, behavioral changes, spirituality
Grob *et al*.^35^	2011	Psilocybin	0.2 mg/kg (oral)	Cancer-related distress	12	Yes	1 day – 4 weeks	Mood, depression, anxiety
Frecska *et al.*^36^	2012	Ayahuasca	583 ± 315.8 mL, 0.73 mg/mL DMT (oral)	Nonclinical	40	Yes	2 days after the 2 weeks	Creativity
Johnson *et al.*^3^	2014	Psilocybin	20 mg/70 kg and/or 30 mg/70 kg (oral)	Nicotine addiction	10	No	1–3 weeks after each session	Mood, substance abuse, attitudes, social effects, behavioral changes, mysticism/spirituality
Bogenschutz *et al.*^37^	2015	Psilocybin	0.3 and 0.4 mg/kg (oral)	Alcohol addiction	10	No	1–4 weeks after each session	Mood, substance abuse
Osório *et al*.^38^	2015	Ayahuasca	2.2 mL/kg, 0.8 mg/mL DMT (oral)	Depression	6	No	1 day – 3 weeks	General psychopathology, depression, mania
Schmid *et al*.^39^	2015	LSD	200 µg (oral)	Nonclinical	16	Yes	1–3 day	Mood, other drug effects
Carhart-Harris *et al*.^40^	2016	LSD	75 µg (intravenous)	Nonclinical	20	Yes	2 weeks	Psychosis, personality
Dolder *et al*.^41, b^	2016	LSD	100 µg (oral)	Nonclinical	24^3^	Yes	1–3 days	Mood, complaints
Ross *et al*.^5^	2016	Psilocybin	0.3 mg/kg (oral)	Cancer-related distress	29	Yes	1 day – 2 weeks	Quality of life, mood, depression, anxiety, attitudes, spirituality, social effects, behavioral changes
Sanches *et al*.^42^	2016	Ayahuasca	2.2 mL/kg, 0.8 mg/mL DMT (oral)	Depression	17	No	1 day – 3 weeks	General psychopathology, depression, mania
Soler *et al*.^43^	2016	Ayahuasca	43.6 (28.8–69.8) mg DMT (oral)	Nonclinical	25	No	1 day	Mindfulness
Sampedro *et al*.^44^	2017	Ayahuasca	148 ± 29 mL, 45 ± 9 mg DMT (oral)	Nonclinical	16	No	1 day	Mindfulness
Carhart-Harris *et al*.^45^	2018	Psilocybin	10 mg and 25 mg (oral)	Depression	20	No	1–3 weeks after 2nd session	General psychopathology, depression, anxiety
Haijen *et al.*^46^	2018	Mixed	n/a	Nonclinical	212–315	No	2–4 weeks	Wellbeing
Lyons and Carhart-Harris^47, c^	2018a	Psilocybin	10 and 25 mg (oral)	Depression	7	Yes	1 week	Attitudes
Lyons and Carhart-Harris^48, c^	2018b	Psilocybin	10 and 25 mg (oral)	Depression	15	Yes	1 week	Attitudes
Schmid and Liechti^49, d^	2018	LSD	200 µg (oral)	Nonclinical	16	Yes	1 month	Mood, anxiety, personality/attitudes, social effects, mysticism, behavioral changes
Soler *et al*.^50^	2018	Ayahuasca	4 sessions, DMT n/a, (oral)	Nonclinical	10	Yes	1 day after the last session	Mindfulness
Stroud *et al*.^51, c^	2018	Psilocybin	10 and 25 mg (oral)	Depression	17	Yes	1 week after the last session	Social effects
Uthaug *et al*.^52^	2018	Ayahuasca	mL n/a, 200 mL: 189–915 mg DMT (oral)	Nonclinical	57	No	1 day–4 weeks	Life satisfaction, depression, anxiety, mindfulness, creativity
Domínguez-Clavé *et al*.^53^	2019	Ayahuasca	n/a (oral)	Nonclinical	45	No	1 day	Mindfulness/emotion regulation
Mason *et al*.^54^	2019	Psilocybin	27.1 mg (oral)	Nonclinical	22–50	No	1–7 days	Life satisfaction, creativity, empathy
Palhano-Fontes *et al*.^4^	2019	Ayahuasca	1 mL/kg, 0.36 ± 0.01 mg/mL of DMT (oral)	Depression	14	Yes	1 day – 1 week	Depression
Uthaug *et al*.^55^	2019	5-MeO-DMT	n/a (inhalation)	Nonclinical	24	No	1 day – 1 month	Life satisfaction, general psychopathology, depression, anxiety, mindfulness, creativity
Anderson *et al*.^56^	2020	Psilocybin	22–32 mg (oral)	AIDS survivor	18	No	3 weeks	Quality of life, general psychopathology, suicidality, depression/grief/demoralization, anxiety, PTSD symptoms, cognitive performance
Barrett *et al*.^57^	2020	Psilocybin	25 mg/70 kg (oral)	Nonclinical	12	No	1 week–1 month	Mood, depression, anxiety, personality
Jiménez-Garrido *et al*.^58^	2020	Ayahuasca	n/a (oral)	Nonclinical	28	No	1 month	Quality of life, general psychopathology, depression, psychosis, personality, acceptance
Murphy-Beiner and Soar^59^	2020	Ayahuasca	n/a (oral)	Nonclinical	48	No	1 day	Mindfulness, flexibility
Netzband *et al*.^60^	2020	Ayahuasca	6 sessions, DMT n/a (oral)	Nonclinical	24	Yes	1 day after last session	Personality
Uthaug *et al*.^61^	2020	5-MeO-DMT	17–61 mg (inhalation)	Nonclinical	11	No	1 week	Depression/stress, anxiety, mindfulness
Zeifman *et al*.^62^	2020	Mixed	n/a	Nonclinical	104	No	2–4 weeks	Suicidality, depression, avoidance
		Mixed	n/a	Nonclinical	254	No	2–4 weeks	Suicidality, depression, avoidance
Davis *et al.*^63^	2021	Psilocybin	20 mg/70 kg and 30 mg/70 kg (oral)	Depression	13	Yes	1–4 weeks	Suicidality, depression, anxiety
Dos Santos *et al.*^64^	2021	Ayahuasca	2 mL/kg, mean 0.68 mg/mL DMT (oral)	Social anxiety	9	Yes	7–21 days	Anxiety
Mans *et al*.^65, e^	2021	Mixed	n/a	Nonclinical	212–315	No	2–4 weeks	Depression, attitudes/personality, acceptance/mindfulness, connectedness/compassion, spirituality
Mason *et al*.^66^	2021	Psilocybin	0.17 mg/kg	Nonclinical	30	Yes	7 days	Creativity
Schindler *et al*.^67^	2021	Psilocybin	0.143 mg/kg	Migraine	10	Yes	2 weeks	Migraines
Uthaug *et al*.^68^	2021	Ayahuasca	7–10 capsules ~552 mg, ~3.6 mg/g DMT, (oral)	Nonclinical	14	Yes	1 day	General psychopathology, depression/stress, anxiety, empathy, mindfulness
Wießner *et al*.^69^	2021	LSD	50 µg (oral)	Nonclinical	24	Yes	1 day–2 weeks	Mindfulness

ROA, route of administration; DMT, N-dimethyltryptamine; LSD, lysergic acid diethylamide; OCD, obsessive-compulsive disorder; PTSD, posttraumatic stress disorder.

a Participants were allocated to one of three experimental groups: 1) LSD + hypnosis + psychotherapy, 2) LSD + psychotherapy 3) LSD.

b Only data from study 1 were reported, and data from study 2 were already reported in Schmid *et al.*^39^

c Subsample of Carhart-Harris *et al*.,^45^ included because new data are presented.

d The same sample as Schmid *et al*.,^39^ included because new data are presented.

e (Sub)sample of Haijen *et al*.,^46^ included because new data are presented.

### Risk of bias

Fourteen of the 48 studies (29%) were characterized by standardization of treatment, a placebo-based control group, and a double-blind allocation (not necessarily fully randomized). Nineteen studies (40%) had a standardized treatment but no control group and/or no double-blind allocation to groups. Fifteen studies (31%) had no standardization of treatment (e.g. observational studies). The classification of individual studies is presented in Supplementary Table S1. While controlled studies investigated predominantly psilocybin and LSD (86%), observational studies explored most often ayahuasca (60%).

### Synthesis of results

#### Subacute effects of psychedelics

**Figure 2.**
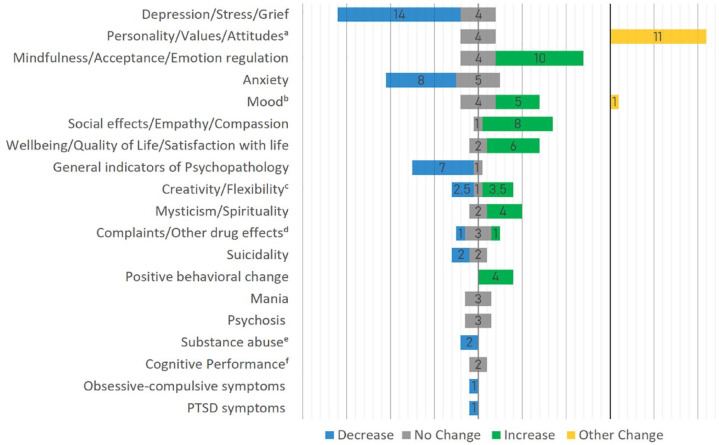
Number of studies reporting a significant effect in the respective outcome domain. ^a^Since the domain of Personality/Values/Attitudes does not qualify for the dichotomous classification of ‘increase/decrease’, all changes were summarized with the label ‘other change’. Nine studies collected data on broad personality measures, e.g. using the Minnesota Multiphasic Personality Inventory,^70^ or the revised NEO Personality Inventory.^71^ Four of those studies (44%) reported subacute effects: one study each reported a decrease in hypochondriasis,^25^ an increase in openness,^40^ an increase in conscientiousness,^57^ and a decrease in neuroticism, and an increase in agreeableness.^60^ Six studies reported on 12 outcome measures assessing specific personality traits/values/attitudes. Except optimism, each of them was assessed only once: an increase was reported in religious values,^23^ optimism,^40,72^ nature relatedness,^47^ absorption, dispositional positive emotions,^57^ self-esteem, emotional stability, resilience, meaning in life, and gratitude.^65^ A decrease was reported in authoritarianism^47^ and pessimism.^48^ Four studies reported on the two subscales ‘attitudes toward life and self’ of the Persisting Effects Questionnaire. All reported increased positive attitudes,^3,5,34,49^ and one study reported increased negative attitudes at low doses of psilocybin.^34^ ^b^Six out of 10 studies reported effects in the outcome domain of mood: one study reported an increase in dreaminess (shown as ‘other change’),^30^ one study reported a subacute decrease in negative affect, tension, depression, and total mood disturbances,^57^ and four studies reported positive mood changes.^3,5,34,49^ ^c^One study observed an increase in convergent and divergent thinking at different subacute assessment points and was therefore classified half as ‘increase’ and half as ‘decrease’.^54^ ^d^Four studies collected complaints in the subacute follow-up using a standardized list of complaints: three of these studies reported no change,^29,39,41^ one study reported an increase in complaints after 1 day but not 1 week.^28^ One other study reported a reduction in migraines.^67^ One study assessed general subjective drug effects lasting into the subacute follow-up period and reported no lasting subjective drug effects.^39^ ^e^Johnson *et al.*^3^ report a peak of withdrawal symptoms 1 week after the substance session. However, since the substance session coincided with the target quit date of tobacco, this was not considered a subacute effect of psilocybin but of tobacco abstinence. ^f^Including intelligence, visual perception,^27^ and a screening for cognitive impairments.^55^

Results for each individual study, classified by the domain of the outcome measures assessed, are displayed in Supplementary Table S1. In combination, all 48 studies covered a total of 19 different psychological outcome domains (% refers to the percentage of studies that included at least one outcome measure of this domain): depression/stress/grief (38%), personality traits/values/attitudes (31%), mindfulness/acceptance/emotion regulation (29%), anxiety (27%), mood (21%), social effects/empathy/compassion (19%), wellbeing/quality of life/life satisfaction (17%), general indicators of psychopathology (17%), creativity/flexibility (15%), mysticism/spirituality (13%), complaints/other drug effects (10%), behavioral change (8%), suicidality (8%), mania (6%), psychosis (6%), substance abuse (4%), cognitive performance (4%), obsessive-compulsive behavior (2%), and PTSD symptoms (2%). Table 2 provides sample characteristics for each of these outcome domains separately. Figure 2 and Supplementary Table S2 provide an overview of the reported subacute effects and their directions, including all findings from the total 4-week subacute follow-up period. Figure S1 summarizes findings separately for three consecutive subacute follow-up periods (1–2 days, 3–14 days, and 15 days–1 month).

**Table 2.** Sample description by outcome domain.

	Count	Years	Follow-up	Study type^a^			Substance						Population		Control group	
				A	B	C	LSD	Psilo	Aya	5-MeO	Mixed	Mesc	Clinical	Non-Cl.	Yes	No
All studies	48	1958–2021	1 day– 1 month	14	19	15	10	16	16	2	3	1	17	31	24	24
	(1774)			(283)	(400)	(1091)	(362)	(265)	(430)	(35)	(673)	(9)	(455)	(1319)	(603)	(1171)
Wellbeing/Quality of Life/Satisfaction with Life	8	2009–2020	1 day– 1 month	1	1	6	–	3	3	1	1	–	2	6	2	6
	(570)			(29)	(18)	(523)		(97)	(134)	(24)	(315)		(47)	(523)	(78)	(492)
Mood	10	1960–2020	1 day– 1 month	7	3	–	3	7	–	–	–	–	4	6	7	3
	(149)			(117)	(32)		(50)	(99)					(61)	(88)	(117)	(32)
General Indicators of Psychopathology	8	2005–2021	1 day– 1 month	–	4	4	–	2	5	1	–	–	4	4	1	7
	(155)				(61)	(94)		(38)	(93)	(24)			(61)	(94)	(14)	(141)
Suicidality	4	2020–2021	1 day– 1 month	1	1	2	–	2	–	–	2	–	2	2	1	3
	(389)			(13)	(18)	(358)		(31)			(358)		(31)	(358)	(13)	(376)
Depression/Stress/Grief	18	1999–2021	1 day– 1 month	4	6	8	–	7	6	2	3	–	8	10	6	12
	(956)			(68)	(81)	(807)		(112)	(136)	(35)	(673)		(129)	(827)	(90)	(866)
Mania	3	1999–2016	1 day– 3 weeks	–	3	–	–	1	2	–	–	–	2	1	1	2
	(31)				(31)			(8)	(23)				(23)	(8)	(8)	(23)
Anxiety	13	1999–2021	1 day– 1 month	5	4	4	1	7	3	2	–	–	6	7	7	6
	(243)			(79)	(58)	(106)	(16)	(112)	(80)	(35)			(92)	(151)	(101)	(142)
Substance Abuse	2	2014–2015	1 week– 1 month	–	2	–	–	2	–	–	–	–	2	–	–	2
	(20)				(20)			(20)					(20)			(20)
Psychosis	3	1999–2020	1 day– 1month	–	2	1	1	1	1	–	–	–	–	3	2	1
	(56)				(28)	(28)	(20)	(8)	(28)					(56)	(28)	(28)
Obsessive-Compulsive Symptoms	1	2006	1 day after each session	–	1	–	–	1	–	–	–	–	1	–	–	1
	(9)				(9)			(9)					(9)			(9)
PTSD Symptoms	1	2020	3 weeks	–	1	–	–	1	–	–	–	–	1	–	–	1
	(18)				(18)			(18)					(18)			(18)
Personality/Values/Attitudes	15	1958–2021	1 day– 1 month	4	8	3	6	5	2	–	1	1	7	8	9	6
	(773)			(153)	(253)	(367)	(313)	(84)	(52)		(315)	(9)	(332)	(441)	(352)	(421)
Mysticism/Spirituality	6	2009–2021	1 week– 1 month	3	1	2	1	3	1	–	1	–	2	4	4	2
	(437)			(63)	(10)	(364)	(16)	(57)	(49)		(315)		(39)	(398)	(112)	(325)
Creativity/Flexibility	7	1964–2021	1 day– 1 month	1	1	5	1	2	3	1	–	–	–	7	3	4
	(264)			(30)	(15)	(219)	(15)	(80)	(145)	(24)				(264)	(85)	(179)
Mindfulness/Acceptance/Emotion Regulation	14	2016–2021	1 day– 1 month	1	3	10	1	–	8	2	3	–	–	14	3	11
	(975)			(24)	(51)	(900)	(24)		(243)	(35)	(673)			(975)	(48)	(927)
Social effects/Empathy/Compassion	9	1964–2021	1 day– 1 month	3	3	3	2	5	1	–	1	–	3	6	6	3
	(484)			(63)	(42)	(379)	(31)	(124)	(14)		(315)		(56)	(428)	(109)	(375)
Positive Behavioral Change	4	2011–2018	1 week– 1 month	3	1	–	1	3	–	–	–	–	2	2	3	1
	(73)			(63)	(10)		(16)	(57)					(39)	(34)	(63)	(10)
Cognitive Performance	2	1971–2020	1 week– 3 weeks	1	1	–	1	1	–	–	–	–	2	–	1	1
	(108)			(90)	(18)		(90)	(18)					(108)		(90)	(18)
Complaints/ Other drug effects	5	1999–2021	1 day– 2 weeks	3	2	–	2	3	–	–	–	–	1	4	4	1
	(66)			(50)	(16)		(40)	(26)					(10)	(56)	(58)	(8)

LSD, lysergic acid diethylamide; PTSD, posttraumatic stress disorder.

The table provides the number of studies and total sample size in brackets.

a Study type: A: standardization of treatment, placebo-based control group, double-blind allocation (not necessarily fully randomized) B: standardization of treatment, no control group and/or no double-blind allocation to groups C: no standardization of treatment (e.g. observational study).

#### Adverse effects

All studies were screened for the reporting of individual-level adverse events. Reports for each study are shown in Supplementary Table S3. Twenty-five studies (52%, with *n* = 550 participants) mentioned whether they assessed adverse events. Of those, 11 studies (44%, with *n* = 297 participants) reported no subacute adverse events, 14 studies (56%, with *n* = 253 participants) reported the following subacute adverse events: *n* = 52 headaches, usually lasting no longer than 1–2 days, *n* = 6 insomnia/sleep disturbances, *n* = 6 tension, *n* = 3 exhaustion, *n* = 3 visual distortion, *n* = 2 fatigue, *n* = 2 migraine, *n* = 2 nausea, *n* = 2 difficulty to concentrate, *n* = 2 vivid dreams, *n* = 1 severe anxiety exacerbation and methamphetamine relapse, *n* = 1 posttraumatic stress flashback, tinnitus, panic, *n* = 1 dry mouth, *n* = 1 altered body sensations, *n* = 1 chest tightness, *n* = 1 physical discomfort, *n* = 1 mild controllable muscle motion, *n* = 1 psychiatric disturbance lasting one week, *n* = 1 no specific description, reversed with therapy. One study reported adverse effects only on the group level and reported an increase of complaints after 1 day but not one week measured with the Vegetative Lability Scale B-L.^28,72^ One study described subacute adverse effects as very low and not significantly different from placebo.^41^ One study described adverse events with no clear distinction between acute and subacute adverse effects^5^ that included nonclinically significant elevations in blood pressure and heart rate, headaches/migraines, nausea, transient anxiety, and transient psychotic-like symptoms (one case of transient paranoid ideation and one case of transient thought disorder).

## Discussion

The present review summarized subacute (i.e., 1 day to 1 month) effects of different classic serotonergic psychedelics on psychological outcome measures and subacute adverse effects. Taken together, the following subacute effects were reported: (1) reductions of psychopathological symptoms (depression, anxiety, suicidality, symptoms related to substance abuse, OCD symptoms, PTSD symptoms), (2) increases in wellbeing/quality of life/life satisfaction, mood, mindfulness/acceptance/emotion regulation, social measures (e.g. relationships and connectedness), mysticism/spirituality, positive behavioral change, (3) mixed changes in personality traits/values/attitudes (e.g. increases in openness, optimism, meaning in life, and gratitude, decreases in authoritarianism), and creativity/flexibility. (4) No changes were reported in manic or psychotic symptoms or cognitive performance measures. (5) Reported adverse events from the subacute window were mild to severe,^73^ including headaches, sleep disturbances, tension, exhaustion, and anxiety exacerbation. No serious subacute adverse events were reported.

The data of this review suggest that psychedelics are associated with subacute effects that outlast the time after acute drug effects have subsided. These include previously reported subacute reductions in depression and anxiety^17,18^ but also a wide range of other effects (see Figure 2). Findings corroborate anecdotal reports of an afterglow phenomenon occurring in the subacute time period after psychedelic substance use comprising predominantly positive effects, including increased wellbeing, reduced psychopathology, and potentially beneficial changes in the perception of self, others, and the environment.^7,9,10^ The frequency and consistency with which subacute effects were observed, however, varied considerably across outcome domains. Most consistently (i.e. in 70–90% of studies that investigated those outcome domains) and within the largest total sample sizes (more than 450 participants), changes were observed in the following domains: depression/stress/grief, mindfulness/acceptance/emotion regulation, personality/values/attitudes, wellbeing/quality of life/life satisfaction, and social effects. Changes in these domains were observed across clinical and nonclinical samples, except for the outcome domain of mindfulness/acceptance/emotion regulation. All studies in this latter domain investigated healthy or unselected convenience samples, allowing no conclusion on the generalizability of this effect to clinical samples. Furthermore, the data on mindfulness/acceptance/emotion regulation and wellbeing/quality of life/life satisfaction predominantly stem from observational studies. By definition, observational studies entail a lower internal validity, for example, through selection biases of study participants, requiring confirmation of findings in more controlled study designs.

For other outcome domains, the consistency of findings across studies was lower (e.g. reductions in anxiety, mood changes, and reduced suicidality). With regard to suicidality and anxiety, floor effects might have contributed to these observations: The absence of suicidal ideation is usually a prerequisite for participation in experimental studies with psychedelics^74^ and the detection of potential reductions in suicidality in laboratory studies therefore limited by low baseline values.^56,63^ Similarly, in four of the five studies that did not observe any subacute effects on anxiety, samples consisted of nonclinical populations with relatively low baseline scores of anxiety. Nevertheless, one of these studies actually observed an effect of time on anxiety that was, however, not specific to the psychedelic group but also present in the placebo group.^68^

For other outcomes, the total sample size was much lower, and sometimes outcomes were collected in just one study (e.g. OCD and PTSD symptoms). These findings are beneficial for generating further hypotheses, but findings should be interpreted with caution before replication in future studies.

Due to its significance for their clinical application, the review focused not only on the intended effects of psychedelics but also summarized data on subacute adverse effects. At the group level, no worsening of psychopathology was reported in any of the studies. Furthermore, there was no evidence of increases in suicidality, manic, or psychotic symptoms, or decreases in cognitive performances – although only a few studies explicitly assessed these outcomes. If changes occurred on the group level, they were directed toward less psychopathology.

Only around half of the studies (52%) mentioned the assessment of individual subacute adverse events. Of those studies, about half reported that they did not observe any subacute adverse events. The other half reported the occurrence of subacute adverse events. If the severity of adverse events was reported, it was mostly mild to moderate. However, not all studies reported severity^73^ and one study observed a severe adverse event (severe anxiety exacerbation).^56^ The subacute adverse event by far most often reported was mild headaches, usually occurring in close temporal proximity to the drug use. Among the less common subacute adverse events were sleep disturbances, tension, and exhaustion. No subacute suicides or death and no full-blown psychotic episodes were reported. These subacute side effects are comparable to the known side effects of selective serotonin reuptake inhibitors (SSRIs),^75^ the drug class most commonly used in treating depression and anxiety. However, the comparison of psychedelics and SSRIs is limited by their different prescription patterns. SSRIs are to be taken daily over extended periods of time, and some of their side effects have been shown to persist during long-term use.^76^ The studies reported here investigated single uses of psychedelics, reporting on transient subacute side effects.

It is important to note, however, that about half of the studies did not specifically describe whether they assessed individual adverse events. And as adverse events were only reported for a subsample of 550 participants, infrequent, rare, or very rare adverse drug reactions may not have been detected.^77^ Furthermore, most studies did not use standardized tools or checklists to assess adverse drug effects. This may result in an underestimation of side effects, as open-ended questions are known to be less sensitive in detecting side effects.^78–80^

In narrative descriptions, subacute effects of psychedelics are usually described as being transient, subsiding gradually after 2–4 weeks.^9^ Interestingly, however, there are very heterogeneous findings on the duration of postacute psychedelic effects. Some of the effects of the subacute period can still be observed in long-term follow-ups. A previous review on the long-term effects of psychedelics reported enduring changes in personality/attitudes, depression, spirituality, anxiety, wellbeing, substance misuse, meditative practices, and mindfulness from 2 weeks up to 4.5 years after psychedelic use.^15^ In a meta-analysis on the effects of psychedelics on depressive symptoms in clinical trials, a rapid and significant reduction of depressive symptoms after psychedelic substance use from day 1 was reported that lasted until the longest follow-up period of 6 months.^18^ We explored the course of subacute effects separately for three subacute time periods (1–2 days, 3–14 days, 15 days–1 month, see Figure S1). Across studies, the majority of subacute effects were observed in all three time frames, although most consistently 3–14 days after the use of psychedelics. Results of the earliest assessment points 1–2 days after the use of psychedelics were most variable, with a greater proportion of statistically nonsignificant findings, especially in the outcome domains of anxiety and mood. An exception to this observation is the outcome domain of creativity/flexibility: increases in creativity were limited to the early subacute time windows. Within single studies with multiple assessment points, various time courses of subacute effects were observed: In the domain of depression/stress/grief, Uthaug *et al.*^55^ observed no decrease in depression and stress 1 day, but 1 month after psychedelic use. Barrett *et al.*,^57^ on the other hand, observed a decrease in stress at week 1, but not at 1 month. Similarly, in the outcome domain of mindfulness, Uthaug *et al.*^52^ observed an increase of certain facets 1 day but not 1 month after psychedelic use, while Uthaug *et al.*^55^ observed an increase at 1 month but not 1 day after psychedelic use. In anxiety, Grob *et al.*^35^ reported no changes in state anxiety and a decrease in trait anxiety not 1 day or 1 week but 1 month after the second treatment session. Similarly, Uthaug *et al.*^55^ reported decreased anxiety not 1 week but 4 weeks after psychedelic use. Barrett *et al.*^57^ also reported a decrease in trait anxiety not 1 week but one month after psychedelic use. However, they additionally observed a decrease in state anxiety 1 week, but not 1 month after psychedelic use. None of the studies that provided multiple subacute assessment points, reported opposing findings (e.g. decreased depression at one and increased depression at another subacute assessment point).

The heterogeneity of findings concerning the duration of subacute effects might be partly explained by considering an increasing relevance of extra-pharmacological factors of action for the preservation of psychedelic effects over time. Even if subacute and long-term effects phenomenologically overlap, underlying mechanisms of actions may shift from transient ‘physiological’ aftereffects to lasting changes in patterns of thought and behaviors after learning and memory processes have taken place. While afterglow effects observed in the subacute window might be correlates of biopsychological remnants of the preceding psychedelic experience that gradually wear off, the transition into long-term effects might depend on individual and environmental resources that support or facilitate the consolidation of initial subacute effects.

In this review, we summarized subacute effects across different classic serotonergic psychedelics. This approach was chosen since classic psychedelics show substantial similarities in their mode of action as agonists at the serotonin 2A receptor and large overlaps in acute effects.^81,82^ However, although the quality of acute experiences is similar across classic psychedelics, duration, and strength of effects may vary based on substance and dosage^81^ and also subacute and long-term effects may differ between different compounds. We therefore explored possible differences in subacute effects between substances. While subacute reductions in the outcome domain of depression/stress/grief were observed across all substances that investigated this outcome domain (psilocybin, ayahuasca, and 5-Meo-DMT), subacute reductions in anxiety were only observed in psilocybin, LSD, and 5-MeO-DMT but not in ayahuasca. However, these comparisons are restricted by a relatively low number of studies per outcome domain and the unequal representation of substances across these domains. Most studies included in this review examined psilocybin (33%), ayahuasca (33%), or LSD (21%), and only a very few studies explored 5-MeO-DMT (4%) or mescaline (2%). Furthermore, the comparison between substances could be distorted by an unequal distribution of study substances across study types and study dates, resulting in varying degrees of internal validity. While 86% of controlled studies administered psilocybin and LSD, 60% of observational trials investigated ayahuasca. Similarly, while 86% of early studies (1958–1972) researched LSD, 78% of modern trials (1999–2021) studied psilocybin or ayahuasca. Findings of this descriptive comparison of different classic psychedelics may therefore inform the development of future hypotheses but will have to be validated using comparative study designs.

### Limitations

The aim of this review was to provide an exhaustive overview of previously reported subacute effects of psychedelics on psychological outcome measures. We therefore decided to include a broad range of studies with different levels of experimental control in the review. As a result, internal validity varied substantially between individual studies, and 72% of the studies lacked a control group, double-blind allocation, and/or standardization of treatment. While the review thus may help to create further hypotheses toward psychedelic drug effects, validity, and reliability of findings, especially those that have only been observed in one or few studies, will have to be confirmed in larger, randomized, and controlled trials.

It is well-known that the acute effects of psychedelics are strongly affected by nonpharmacological context variables.^83^ Most studies in this review were either laboratory studies or observational studies of psychedelic ceremonies. Both contexts usually provide a safe environment, and at least in modern trials, there is a minimum standard of preparation (e.g. detailed information during informed consent) and aftercare that may even extend up to several therapeutic sessions surrounding the day of drug administration (e.g. screening for adverse events, and ‘integration’ of experiences into everyday life).^74,84,85^ Results of this review can therefore not be generalized to uncontrolled and recreational contexts of drug administration where unpleasant and challenging experiences might not be cushioned by a holding environment. This aspect is particularly important to consider when assessing the adverse events observed in this review. While there was no evidence for manic or psychotic subacute symptoms in the studies that were included in this review, there are reports of such subacute effects, particularly in older studies and case reports that did not meet our inclusion criteria.^86,87^ Furthermore, selective reporting or publication bias must be considered as another reason for the predominantly positive effects. Although this is a problem of the scientific community in general, it previously has been discussed whether personal overinvolvement and increased public interests in psychedelic research may pose this field at an increased risk of underreporting null or negative findings and biasing research toward more positive subacute effects.^88,89^ Before replication in larger, preregistered, and controlled studies, results of this review should therefore not be considered to represent the average of expected subacute effects, but a summary of previously observed effects under favorable conditions.

### Implications for future research

The present review summarized effects of psychedelics that were observed at any time during a 1-month subacute period. As discussed earlier, there are, however, heterogeneous findings on the duration of subacute effects. Future studies are needed to elaborate on the time course of specific subacute symptoms and the role of possible moderators for the quality and intensity of subacute or afterglow effects. These moderators may include substance characteristics (e.g. different psychedelic substances and dosages), experiential aspects of the acute psychedelic experiences, sample characteristics (e.g. clinical *versus* nonclinical populations), or environmental factors (e.g. the combination of psychedelic substance use with social support and/or psychotherapy). The use of ecological momentary assessment tools (e.g. brief daily surveys on the participant’s mobile phone), for example, would allow to track and compare the course of subacute effects more accurately and at a higher resolution. For a time-efficient screening of subacute effects, it would be furthermore helpful to have an instrument, specifically designed to capture different aspects of the afterglow phenomenon, similar to the standard questionnaires used to assess acute effects of psychedelics.^90^ This would allow us to compare different substances and dosages more easily, for example, by expanding databases like ‘The Altered States Database’.^91^

Precise knowledge of the progression of postacute effects could help to optimize the clinical application of psychedelics, for example, through the augmentation of drug effects with extra-pharmacological interventions. It has long been hypothesized that the subacute ‘afterglow’ period opens a window of enhanced effectivity of psychotherapeutic interventions,^9^ and intensified psychotherapeutic work in the subacute period may allow people to maintain beneficial effects even beyond the early days and weeks after psychedelic use.^92^ In modern clinical trials, postsession meetings in close temporal proximity to the acute experience can already be considered part of the standard study protocol.^74^ Their focus has been on safety and harm reduction, as well as meaning-making and ‘integration’ of experiences into everyday life, which has been described as helpful to prolong therapeutic benefits.^74,84,85^ However, to date, no evidence for the effectivity of such ‘integration’ sessions to prolong the beneficial effects of psychedelics is available, and no systematic research on the optimal design of these postsessions (e.g. number of postsessions, time interval between psychedelic use and postsessions, or content) has been conducted. Future studies are needed to explore the basis of a successful transition of subacute into beneficial long-term effects and to assess whether nonpharmacological interventions might be helpful to support this process.

## Conclusion

If subacute effects occurred after using psychedelics in a safe environment, these were, for many participants, changes toward indicators of increased mental health and wellbeing. The use of psychedelics was associated with a range of subacute effects that corroborate narrative reports of a subacute afterglow phenomenon, comprising reduced psychopathology, increased wellbeing, and potentially beneficial changes in the perception of self, others, and the environment. Mild-to-severe subacute adverse events were observed, including headaches, sleep disturbances, and individual cases of increased psychological distress, no serious adverse event was reported. Since many studies lacked a standardized assessment of adverse events, results might be biased, however, by selective assessment or selective reporting of adverse effects and rare or very rare adverse effects may not have been detected yet due to small sample sizes.

Future studies are needed to investigate the role of possible moderator variables (e.g. different psychedelic substances and dosages), the relationship between acute, subacute, and long-term effects, and whether and how the consolidation of positive effects from the subacute window into long-term mental health benefits can be supported.

## Supplemental Material

sj-docx-1-tpp-10.1177_20451253231172254 – Supplemental material for The psychedelic afterglow phenomenon: a systematic review of subacute effects of classic serotonergic psychedelics Click here for additional data file. Supplemental material, sj-docx-1-tpp-10.1177_20451253231172254 for The psychedelic afterglow phenomenon: a systematic review of subacute effects of classic serotonergic psychedelics by Ricarda Evens, Marianna Elisa Schmidt, Tomislav Majić and Timo Torsten Schmidt in Therapeutic Advances in Psychopharmacology

## Declarations

**Ethics approval and consent to participate:** Not applicable.

**Consent for publication:** Not applicable.

**Availability of data and materials:** Not applicable.
