# Supplementary material for: The psychedelic afterglow phenomenon: a systematic review of subacute effects of classic serotonergic psychedelics
Source: Ther Adv Psychopharmacol. 2023 May 29;13:20451253231172254. doi: 10.1177/20451253231172254 (PMC10240558; doi:10.1177/20451253231172254)
Supplement: sj-docx-1-tpp-10.1177_20451253231172254 – Supplemental material for The psychedelic afterglow phenomenon: a systematic review of subacute effects of classic serotonergic psychedelics [file sj-docx-1-tpp-10.1177_20451253231172254.docx]

**Supplementary material**

[**Search terms**](#_Toc128760213)

[**Table S1: Subacute effects of individual studies by outcome domain**](#_Toc128760214)

[**Table S2: Frequency and total sample size of studies that reported subacute effects by outcome domain**](#_Toc128760215)

[**Figure S2: Subacute effects across time**](#_Toc128760216)

[**Table S4: Subacute adverse events of individual studies**](#_Toc128760217)

# Search terms

**PubMed:**

(((((((((((((((((((LSD) OR lysergic acid diethylamide) OR Psilocybin) OR Mescaline) OR Ayahuasca) OR Dimethyltryptamin*) OR n,n-dimethyltryptamin*) OR DMT) OR 5-Methoxy-N, N-dimethyltryptamin*) OR 5-MeO-DMT) OR 5meodmt) AND (((((((subacute) OR sub-acute) OR sub acute) OR post acute) OR post-acute) OR afterglow) OR ((((psychology) OR psychiatry) OR psychotherapy) OR neuroscience))))) NOT (((((((((mouse[Title]) OR mice[Title] OR rat[Title]) OR rats[Title]) OR rodent[Title]) OR rodents[Title]) OR cats [Title]) OR cat[Title]) OR primate[Title]) OR primates[Title]))))))

**Web of Science:**

(ALL=(lsd) OR ALL=(lysergic acid diethylamide) OR (ALL=(lysergic) AND ALL=(acid) AND ALL=(diethylamide)) OR ALL= (psilocybin) OR ALL=(mescaline) OR ALL=(banisteriopsis) OR ALL=(ayahuasca) OR ALL=(dmt) OR ALL= (dimethyltryptamin) OR ALL=(dimethyltryptamine) OR ALL=(dimethyltryptamines) OR ALL=(dimethyltryptamins) OR ALL=(n, n dimethyltryptamin) OR ALL=(n, n dimethyltryptamine) OR ALL=(n, n dimethyltryptamines) OR ALL=(5 methoxy n, n dimethyltryptamin) OR ALL=(5 methoxy n, n dimethyltryptamine) OR ALL=(5 methoxy n, n dimethyltryptamins) OR ALL=(methoxydimethyltryptamines) OR ALL=(5 meo dmt) OR ALL=(5meodmt)) AND (((ALL=(subacute) OR ALL=(sub-acute)) OR (ALL=(sub) AND ALL=(acute)) OR ((ALL=(post) AND ALL=(acute)) OR ALL=(post-acute)) OR ALL=(afterglow)) OR (ALL=(psychology) OR ALL=(psychiatry) OR ALL=(psychotherapy) OR ALL=(neurosciences) OR ALL=(neuroscience))) NOT (TI=(mouse) OR TI=(mice) OR TI=(rat) OR TI=(rats) OR TI=(rodent) OR TI=(rodents) OR TI=(cats) OR TI=(cat) OR TI=(primate) OR TI=(primates))

| Table S1: Subacute effects of individual studies by outcome domain | | | | | | | | | | | | | | | |  |
| --- | --- | --- | --- | --- | --- | --- | --- | --- | --- | --- | --- | --- | --- | --- | --- | --- |
| **Reference** | **Year** | **Study  type** | **Substance** | **Dosage  (form of administration)** | **Population** | **Sample size** | **Age  (range or SD)** | **Sex female  (%)** | **Control group** | **Subacute  follow-up** | **Instrument** | **Abbreviation** | **Results^a^** | **Summary^b^** | **Comments** |  |
| **Wellbeing/Quality of Life/Satisfaction with life** | | | | | | | | | | | | | | | |  |
| Trichter et al. | (2009) | C | Ayahuasca | n/a | Ceremony-participants, ayahuasca-naïve | 49 | 33 (n/a) | 26 (53) | between group: no drug | 1 week, 4 weeks | Spiritual Well-Being Scale | SWB | No change | ± |  |  |
| Ross et al. | (2016) | A | Psilocybin | 0.3 mg/kg (oral) | Patients with cancer-related anxiety and depression | 29 | 56 (22-75) | 18 (62) | crossover with 250mg niacin | 2 weeks | WHO Quality of Life-Bref | WHOQOL-Bref | Increase in physical health, psychological and environment subscales, but not in social relationships | +/± |  |  |
|  |  |  |  |  |  |  |  |  |  |  | Functional Assessment of Chronic Illness Therapy-Spiritual Well-Being | FACIT-SWB | Increase in all subscales |  |  |  |
| Haijen et al. | (2018) | C | Mixed | n/a | Volunteers participating in online survey | 212-315 | 29 (10.4)^1^ | 165 (25)^1^ | none | 2 weeks,  4 weeks | Warwick-Edinburgh Mental Wellbeing Scale | WEMWBS | Increase at both time points | + | ^1^Data from baseline sample size n = 654 |  |
| Uthaug et al. | (2018) | C | Ayahuasca | Quantity n/a, per 200 mL: 189.4-915.4 mg DMT, 485.5-1261.7 mg harmine, and 38.1-892.0 mg harmaline (oral) | Ceremony-participants | 57 | n/a | 36 (63) | none | 1 day, 4 weeks | Satisfaction with Life Scale | SWLS | Increase at 1 day, but not 1 month | +/± |  |  |
| Mason et al. | (2019) | C | Psilocybin | Mean 27.1 mg psilocin (oral) | Participants of psilocybin retreats | 22-50 | 35 (8.9)^1^ | 26 (47)^1^ | none | 1 day, 7 days | Satisfaction with Life Scale | SWLS | Increase at both time points | + | ^1^Of the baseline sample of n = 55 |  |
| Uthaug et al. | (2019) | C | 5-MeO-DMT | Dose n/a (inhalation) | Participants of recreational 5-MeO-DMT retreats | 24 | 38 (SEM = 0.8)^1^ | 17 (40)^1^ | none | 1 day, 1 month | Satisfaction with Life Scale | SWLS | Increase at both time points | + | ^1^Data of baseline sample n = 42 |  |
| Anderson et al. | (2020) | B | Psilocybin | 22-32 mg (oral) | Demoralized older self-identified gay AIDS survivor men | 18 | 59.2 (4.4) | 0 (0) | none | 3 weeks | McGill Quality of Life Questionnaire-Revised (overall) | MQoL-R | Increase | + |  |  |
| Jiménez-Garrido et al. | (2020) | C | Ayahuasca | n/a (oral) | Ceremony-participants, ayahuasca-naïve | 28 | 35 (20-65)^1^ | 28 (70)^1^ | none | 1 month | WHO Quality of Life-Bref | WHOQOL-Bref | No change | ± | ^1^Data of baseline sample n = 40 (only study 1) |  |
|  |  |  |  |  |  |  |  |  |  |  | Medical Outcomes Study 36-item Short-Form | SF-36 | No change |  |  |  |
| **Mood** | | | | | | | | | | | | | | | |  |
| Lebovits et al. | (1960) | A | LSD | 100 µg (oral) | Healthy volunteers, paid medical students | 10 | n/a | 2 (20) | crossover with 15mg JB 318 | 3-9 days | Clyde Mood Scale |  | No change | ± |  |  |
| Hasler et al. | (2004) | A | Psilocybin | 45, 115, 215, and 315 µg/kg in randomized order, each two weeks apart (oral) | Healthy volunteers | 8 | 30 (22-44) | 4 (50) | crossover with lactose | 1 day after each session | Adjective Mood Rating Scale | AMRS | No changes except increased dreaminess after the highest psilocybin dose | +/± |  |  |
| Griffiths et al. | (2011) | A | Psilocybin | 4 sessions: 5, 10, 20, 30 mg/70 kg, in ascending or descending order, each one month apart (oral) | Healthy volunteers, Hallucinogen-naïve | 18 | 46 (29-62) | 10 (56) | crossover with placebo | 3-4 weeks after each session | Persisting Effects Questionnaire (mood) | PEQ | Increasing effects as a function of dose in positive subscale. Negative ratings did not differ. | + |  |  |
| Grob et al. | (2011) | A | Psilocybin | 0.2 mg/kg (oral) | Patients with advanced-stage cancer and anxiety | 12 | n/a (36-58) | 11 (92) | crossover with 250 mg niacin | 1 day,  2 weeks,  4 weeks | Profile of mood states-Brief | POMS | No change | ± |  |  |
| Johnson et al. | (2014) | B | Psilocybin | 2-3 sessions: 20 mg/70 kg and/or 30 mg/70 kg, 2 and 5 weeks apart (oral) | Nicotine-dependent smokers, otherwise psychiatrically healthy | 10 | 51 (26-65) | 5 (33) | none | 1 week | Persisting Effects Questionnaire (mood) | PEQ | Greater positive than negative effects. | + |  |  |
| Bogenschutz et al. | (2015) | B | Psilocybin | 0.3 and 0.4 mg/kg, 4 weeks apart (oral) | Patients with active alcohol-dependency | 10 | 40 (25-56) | 4 (40) | none | 1 week,  4 weeks | Profile of mood states | POMS | No change | ± |  |  |
| Schmid et al. | (2015) | A | LSD | 200 µg (oral) | Healthy volunteers | 16 | 29 (25-51) | 8 (50) | crossover with placebo | 1 day | Adjective Mood Rating Scale | AMRS | No change | ±/+ |  |  |
| Schmid & Liechti^1^ | (2018) |  |  |  |  |  |  |  |  | 1 month | Persisting Effects Questionnaire (mood) | PEQ | Increases in positive subscale. No changes in negative subscale. Compared with assumption of no change (rating = 0). |  | ^1^Same sample as Schmidt et al. 2015 |  |
| Dolder et al. | (2016) | A | LSD | 100 µg (oral) | Healthy volunteers, mostly hallucinogen-naive | 24^1^ | 33 (25-60) | 12 (50) | crossover with placebo | 1 day | Adjective Mood Rating Scale | AMRS | No change | ± | ^1^Only data from study 1 reported, data of study 2 already reported in Schmid et al. 2015 |  |
| Ross et al. | (2016) | A | Psilocybin | 0.3 mg/kg (oral) | Patients with cancer-related anxiety and depression | 29 | 56 (22-75) | 18 (62) | crossover with 250mg niacin | 2 weeks | Persisting Effects Questionnaire (mood) | PEQ | Increases in positive subscale. No changes in negative subscale. | + |  |  |
|  |  |  |  |  |  |  |  |  |  |  |  |  |  |  |  |  |
|  |  |  |  |  |  |  |  |  |  |  |  |  |  |  |  |  |
| Barrett et al. | (2020) | B | Psilocybin | 25 mg/70 kg (oral) | Healthy volunteers | 12 | 32 (7.5) | 7 (58) | none | 1 week, 1 month | Positive and Negative Affect Scale – X | PANAS-X | Decrease in negative affect subscale at both time points. Other scales not reported. | +/± |  |  |
|  |  |  |  |  |  |  |  |  |  |  | Profile of Mood States | POMS | Decreases: tension, depression, and total mood disturbance at 1 week  No change at 1 month |  |  |  |
| **General indicators of Psychopathology** | | | | | | | | | | | | | | | |  |
| Barbosa et al. | (2005) | C | Ayahuasca | n/a (oral) | Ceremony-participants, ayahuasca-naïve | 28 | 36 (18-56) | 12 (43) | none | 7-14 days | Clinical Interview Schedule-Revised Edition | CIS-R | Decrease in minor psychiatric symptoms | - |  |  |
| Osório et al. | (2015) | B | Ayahuasca | 2.2 mL/kg with 0.8 mg/mL DMT, 0.21 mg/mL harmine (oral) | Patients with recurrent MDD | 6 | 44 (13.6) | 4 (67) | none | 1 day 1 week 2 weeks 3 weeks | Brief Psychiatric Rating Scale | BPRS | Decrease: Anxious-Depression. No changes: withdrawal-retardation, thinking disorder, activation | -/± |  |  |
|  |  |  |  |  |  |  |  |  |  |  |  |  |  |  |  |  |
| Sanches et al. | (2016) | B | Ayahuasca | 2.2 mL/kg, 0.8 mg/mL DMT, 0.21 mg/mL harmine, no harmaline (oral) | Patients with recurrent MDD | 17 | 43 (12.11) | 14 (82) | none | 1 day 1 week 2 weeks 3 weeks | Brief Psychiatric Rating Scale | BPRS | Decrease: Anxious-Depression, Thinking Disorder (not 1 week), Withdrawal-Retardation  No change: Activation subscale | -/± |  |  |
| Carhart-Harris et al. | (2018) | B | Psilocybin | 10 and 25 mg, 1 week apart (oral) | Patients with treatment resistant MDD | 20 | 44 (27-64) | 6 (30) | none | 1 week after 2nd session | Global Assessment of Functioning | GAF | Increased | - |  |  |
| Uthaug et al. | (2019) | C | 5-MeO-DMT | n/a (inhalation) | Participants of recreational 5-MeO-DMT retreats | 24 | 38 (SEM = 0.8)^1^ | 17 (40)^1^ | none | 1 day, 1 month | Brief Symptom Inventory 18 | BSI-18 | No change at 1 day Decreases in all subscales at 4 weeks: depression, anxiety, somatization | -/± | ^1^data of baseline sample n = 42 |  |
| Anderson et al. | (2020) | B | Psilocybin | 22-32 mg (oral) | Demoralized older self-identified gay AIDS survivor men | 18 | 59.2 (4.4) | 0 (0) | none | 3 weeks | Clinical Global Impression-Severity | CGI-S | Decrease | - |  |  |
| Jiménez-Garrido et al. | (2020) | C | Ayahuasca | n/a (oral) | Ceremony-participants, ayahuasca-naïve | 28 | 35 (20-65)^1^ | 28 (70)^1^ | none | 1 month | Symptom Check-List-90-Revised | SCL-90-R | Decrease: anxiety and hostility,  No change: all other subscales | -/± | ^1^ data of baseline sample n = 40 (only study 1) |  |
|  |  |  |  |  |  |  |  |  |  |  |  |  |  |  |  |  |
| Uthaug et al. | (2021) | C | Ayahuasca | 7-10 capsules 552mg ± 66.3 with of 3.6 ± 0.2 mg/g DMT, 10.1 mg/g ± 0.8 harmine, 0.7 ± 0.1 harmaline (mean ± SD) | Students of "ayahuasca school" | 14 | 40.2 (10.1)^1^ | 18 (60)^1^ | Between group: Placebo | 1 day | Brief Symptom Inventory 18 | BSI-18 | Decrease in anxiety pre to post in ayahuasca and placebo group, no changes in depression and somatization | ± | ^1^data of total sample (including control group) |  |
| **Specific Psychopathology** | | | | | | | | | | | | | | | |  |
| **Suicidality** |  |  |  |  |  |  |  |  |  |  |  |  |  |  |  |  |
| Anderson et al. | (2020) | B | Psilocybin | 22-32 mg (oral) | Demoralized older self-identified gay AIDS survivor men | 18 | 59.2 (4.4) | 0 (0) | none | 1 day,  1,2,3 weeks | Columbia Suicidality Severity Rating Scale | C-SSRS | No change | ± |  |  |
|  |  |  |  |  |  |  |  |  |  | 3 weeks | Schedule of Attitudes towards Hastened Death | SAHD |  |  |  |  |
| Zeifman et al. | (2020) | C | Mixed | n/a | Volunteers in online survey intending to take a psychedelic | 104 | 29.28 (9.94) | 31 (30) | none | 2 weeks,  4 weeks | Suicidal Ideation Attributes Scale + QUIDS SI item | SIDAS+QIDS-SI | Decrease in suicidal ideation at both follow-ups. Significant reduction of suicial ideation from 2-weeks to 4-weeks. | - |  |  |
|  |  |  |  |  | Volunteers in online survey intending to attended psychedelic ceremony | 254 | 43.61 (12.46) | 115 (45) | none | 4 weeks | Suicidal Ideation Attributes Scale + QUIDS SI item | SIDAS+QIDS-SI | Decrease | - |  |  |
| Davis et al. | (2021) | A | Psilocybin | 20mg/70kg and 30mg/70kg, 1.6 weeks apart (oral) | Patients with moderate to severe MDD | 13 | 43.6 (13.0) | 9 (69) | Waiting list | 1 weeks,  4 weeks | Columbia Suicidality Severity Rating Scale | C-SSRS | No change compared to control group | ± |  |  |
| **Depression/Stress/Grief** | | | | | | | | | | | | | | | |  |
| Gouzoulis-Mayfrank et al. | (1999) | B | Psilocybin | 0.2mg/kg but not more than 15mg total dose (oral) | Healthy volunteers: physicians or psychologists with scientific or clinical interest in study | 8 | 31 (29-37) | 5 (63) | between group: 0.2 mg/kg d-amphetamine, or 2 mg/kg MDE, or placebo | 1 day,  7 days | Bech-Rafaelsen Melancholia Scale | BRMES | No change | ± |  |  |
| Grob et al. | (2011) | A | Psilocybin | 0.2 mg/kg (oral) | Patients with advanced-stage cancer and anxiety | 12 | n/a (36-58) | 11 (92) | crossover with 250 mg niacin | 1 day,  2 weeks,  4 weeks | Beck Depression Inventory | BDI | No change | ± |  |  |
|  |  |  |  |  |  |  |  |  |  |  |  |  |  |  |  |  |
|  |  |  |  |  |  |  |  |  |  |  |  |  |  |  |  |  |
| Osório et al. | (2015) | B | Ayahuasca | 2.2 mL/kg with 0.8 mg/mL DMT, 0.21 mg/mL harmine (oral) | Patients with recurrent MDD | 6 | 44 (13.6) | 4 (67) | none | 1 day 1 week 2 weeks 3 weeks | Hamilton Rating Scale for Depression | HAM-D | MADRS:Decrease at all subacute time points  HAM-D: Decrease at day 1, 7, 21 but not at day 14 | -/± |  |  |
|  |  |  |  |  |  |  |  |  |  |  | Montgomery-Åsberg Depression Rating Scale | MADRS |  |  |  |  |
| Ross et al. | (2016) | A | Psilocybin | 0.3 mg/kg (oral) | Patients with cancer-related anxiety and depression | 29 | 56 (22-75) | 18 (62) | crossover with 250mg niacin | 1 day,  2 weeks | Beck Depression Inventory | BDI | Decrease in all measures and all available subacute time points only after psilocybin not after placebo | - | ^1^Data available only for 2 week follow-up |  |
|  |  |  |  |  |  |  |  |  |  |  | Hospital Anxiety and Depression Scale (Depression) | HADS-D |  |  |  |  |
|  |  |  |  |  |  |  |  |  |  |  | Demoralization Scale^1^ | DEM |  |  |  |  |
|  |  |  |  |  |  |  |  |  |  |  | Hopelessness Assessment and Illness scale^1^ | HAI |  |  |  |  |
| Sanches et al. | (2016) | B | Ayahuasca | 2.2 mL/kg with 0.8 mg/mL DMT, 0.21 mg/mL harmine, no harmaline (oral) | Patients with recurrent MDD | 17 | 43 (12.11) | 14 (82) | none | 1 day 1 week 2 weeks 3 weeks | Hamilton Rating Scale for Depression | HAM-D | Decrease in all measures and all available subacute time points | - |  |  |
|  |  |  |  |  |  |  |  |  |  |  | Montgomery-Åsberg Depression Rating Scale | MADRS |  |  |  |  |
| Carhart-Harris et al. | (2018) | B | Psilocybin | 2 sessions: 10 mg and 25 mg, 1 week apart (oral) | Patients with treatment resistant MDD | 20 | 44 (27-64) | 6 (30) | none | 1 week 2 weeks 3 weeksafter 2nd session | Quick Inventory of Depressive Symptoms | QIDS | Decrease in all measures^2^ and all available subacute time points | - | ^1^Data available only for 1 week follow-up |  |
|  |  |  |  |  |  |  |  |  |  |  | Hamilton Rating Scale for Depression^1^ | HAM-D |  |  | ^2^in Carhart-Harris et al. (2016). Psilocybin with psychological support for treatment-resistant depression: An open-label feasibility study. The Lancet Psychiatry, 3(7), 619–627. https://doi.org/10.1016/S2215-0366(16)30065-7  additionally reported: decrease in Montgomery-Åsberg Depression Rating Scale MADRS |  |
|  |  |  |  |  |  |  |  |  |  |  | Beck Depression Inventory^1^ | BDI |  |  |  |  |
|  |  |  |  |  |  |  |  |  |  |  | Snaith Hamilton Pleasure Scale^1^ | SHAPS |  |  |  |  |
| Uthaug et al. | (2018) | C | Ayahuasca | Quantity n/a, per 200 mL: 189.4-915.4 mg DMT, 485.5-1261.7 mg harmine, and 38.1-892.0 mg harmaline (oral) | Ceremony-participants | 57 | n/a | 36 (63) | none | 1 day, 4 weeks | Depression, Anxiety, and Stress Scale-21 (Stress+Depression) | DASS-21 | Decrease of stress and depression at all subacute time points | - |  |  |
| Palhano-Fontes et al. | (2019) | A | Ayahuasca | 1 mL/kg with 0.36 ± 0.01 mg/mL of DMT, 1.86 ± 0.11 mg/mL of harmine, 0.24 ± 0.03 mg/mL of harmaline, and 1.20 ± 0.05 mg/mL of tetrahydroharmine (mean ± SD) (oral) | Patients with treatment resistant MDD | 14 | 40 (19-56) | 11 (79) | between group: placebo | 1 day, 2 days, 1 week | Hamilton Rating Scale for Depression^1^ | HAM-D | Decrease in all measures and all available subacute time points only after ayahuasca not after placebo | - | ^1^Data available only for 1 week follow-up |  |
|  |  |  |  |  |  |  |  |  |  |  | Montgomery-Åsberg Depression Rating Scale | MADRS |  |  |  |  |
| Uthaug et al. | (2019) | C | 5-MeO-DMT | Dose n/a (inhalation) | Participants of recreational 5-MeO-DMT retreats | 24 | 38 (SEM = 0.8)^1^ | 17 (40)^1^ | none | 1 day, 1 month | Depression, Anxiety, and Stress Scale 21 (Depression, Stress) | DASS-21 | No change at 1 day Decreases in depression and stress at 1 month | -/± | ^1^Data of baseline sample n = 42 |  |
| Anderson et al. | (2020) | B | Psilocybin | 22-32 mg (oral) | Demoralized older self-identified gay AIDS survivor men | 18 | 59.2 (4.4) | 0 (0) | none | 3 weeks | Demoralization Scale II | DS-II | Decrease | - |  |  |
|  |  |  |  |  |  |  |  |  |  |  | Center for Epidemiological Studies Depression Scale-Revised | CESD-R | Decrease |  |  |  |
|  |  |  |  |  |  |  |  |  |  |  | Inventory of Complicated Grief-Revised | ICG-R | Decrease |  |  |  |
| Barrett et al. | (2020) | B | Psilocybin | 25 mg/70 kg (oral) | Healthy volunteers | 12 | 32 (7.5) | 7 (58) | none | 1 week, 1 month | Depression, Anxiety, Stress Scale-21 items (Stress) | DASS-21 | Decrease in stress at 1 week, but not at 1 month Depression scale: not reported | -/± |  |  |
| Jiménez-Garrido et al. | (2020) | C | Ayahuasca | n/a (oral) | Ceremony-participants, ayahuasca-naïve | 28 | 35 (20-65)^1^ | 28 (70)^1^ | none | 1 month | Hamilton Depression Rating Scale | HAM-D | No change | ± | ^1^Data of baseline sample n = 40 (only study 1) |  |
| Uthaug et al. | (2020) | C | 5-MeO-DMT | Single dose 3-24 mg, summed total dose 17-61 mg (inhalation) | Participants of 5-MeO-DMT one-on-one-sessions | 11 | 33 (8.59) | 3 (27) | none | 1 week | Depression, Anxiety, Stress Scale-21 items (Stress, Depression) | DASS-21 | Decrease in stress No change in depression | -/± |  |  |
|  |  |  |  |  |  |  |  |  |  |  |  |  |  |  |  |  |
|  |  |  |  |  |  |  |  |  |  |  |  |  |  |  |  |  |
| Zeifman et al. | (2020) | C | Mixed | n/a | Volunteers in online survey intending to take a psychedelic | 104 | 29.28 (9.94) | 31 (30) | none | 2 weeks,  4 weeks | Quick Inventory of Depressive Symptoms | QIDS | Reduction in depression severity at both follow-ups | - |  |  |
|  |  |  |  |  | Volunteers in online survey intending to attended psychedelic ceremony | 254 | 43.61 (12.46) | 115 (45) | none | 2 weeks,  4 weeks | Quick Inventory of Depressive Symptoms | QIDS | Reduction in depression severity at both follow-ups | - |  |  |
| Davis et al. | (2021) | A | Psilocybin | 2 sessions, 20mg/70kg and 30mg/70kg, 1.6 weeks apart (oral) | Patients with moderate to severe MDD | 13 | 43.6 (13.0) | 9 (69) | between group: waiting list | 1 weeks,  4 weeks | Hamilton Depression Rating Scale | GRID-HAM-D | Greater reduction in depression scores in the immediate treatment condition compared with waiting control group | - |  |  |
|  |  |  |  |  |  |  |  |  |  |  | Beck Depression Inventory | BDI |  |  |  |  |
|  |  |  |  |  |  |  |  |  |  |  | Quick Inventory of Depressive Symptomatology | QIDS-SR |  |  |  |  |
|  |  |  |  |  |  |  |  |  |  |  | Patient Health Questionnaire – 9 item^1^ | PHQ-9 |  |  | ^1^Only available for week 4 |  |
| Mans et al. | (2021) | C | Mixed | n/a | Volunteers participating in online survey | 212-315 | 28.9 (10.45) | 165 (25)^1^ | none | 2 weeks,  4 weeks | Quick Inventory of Depression Symptoms, 16-item Self Report | QIDS-SR16 | Decrease | - | ^1^Data from baseline sample size n = 654. Data are (sub-)sample of Haijen et al., 2018, included because new data are presented |  |
| Uthaug et al. | (2021) | C | Ayahuasca | 7-10 capsules 552mg ± 66.3 with of 3.6 ± 0.2 mg/g DMT, 10.1 mg/g ± 0.8 harmine, 0.7 ± 0.1 harmaline (mean ± SD) | Students of "ayahuasca school" | 14 | 40.2 (10.1)^1^ | 18 (60)^1^ | Between group: Placebo | 1 day | Depression, Anxiety, and Stress Scale 21 (Stress, Depression) | DASS-21 | Decrease in stress and depression pre to post in ayahuasca and placebo group; however, the reduction in symptoms of depression was stronger in the placebo group. | ± | ^1^Data of total sample (including control group) |  |
| **Mania** | | | | | | | | | | | | | | | |  |
| Gouzoulis-Mayfrank et al. | (1999) | B | Psilocybin | 0.2 mg/kg but not more than 15 mg total dose (oral) | Healthy volunteers: physicians or psychologists with scientific or clinical interest in study | 8 | 31 (29-37) | 5 (63) | between group: 0.2 mg/kg d-amphetamine, or 2 mg/kg MDE, or placebo | 1 day,  7 days | Bech-Rafaelsen Mania Scale | BRMAS | No change | ± |  |  |
| Osório et al. | (2015) | B | Ayahuasca | 2.2 mL/kg, 0.8 mg/mL DMT, 0.21 mg/mL harmine (oral) | Patients with recurrent MDD | 6 | 44 (13.6) | 4 (67) | none | 1 day 1 week 2 weeks 3 weeks | Young Mania Rating Scale | YMRS | No change | ± |  |  |
| Sanches et al. | (2016) | B | Ayahuasca | 2.2 mL/kg, 0.8 mg/mL DMT, 0.21 mg/mL harmine, no harmaline (oral) | Patients with recurrent MDD | 17 | 43 (12.11) | 14 (82) | none | 1 day 1 week 2 weeks 3 weeks | Young Mania Rating Scale | YMRS | No change | ± |  |  |
| **Anxiety** | | | | | | | | | | | | | | | |  |
| Gouzoulis-Mayfrank et al. | (1999) | B | Psilocybin | 0.2mg/kg but not more than 15mg total dose (oral) | Healthy volunteers: physicians or psychologists with scientific or clinical interest in study | 8 | 31 (29-37) | 5 (63) | between group: 0.2 mg/kg d-amphetamine, or 2 mg/kg MDE, or placebo | 1 day,  7 days | Spielberger’s State-Trait Anxiety Inventory (state version) | STAI | No change | ± |  |  |
| Grob et al. | (2011) | A | Psilocybin | 0.2 mg/kg (oral) | Patients with advanced-stage cancer and anxiety | 12 | n/a (36-58) | 11 (92) | crossover with 250 mg niacin | 1 day,  2 weeks,  4 weeks | Spielberger’s State-Trait Anxiety Inventory (state + trait version) | STAI | No changes in STAI state  Decrease in STAI trait at 1 month after second treamtent session. | -/± |  |  |
| Ross et al. | (2016) | A | Psilocybin | 0.3 mg/kg (oral) | Patients with cancer-related anxiety and depression | 29 | 56 (22-75) | 18 (62) | crossover with 250mg niacin | 1 day,  2 weeks | Hospital Anxiety and Depression Scale (Anxiety) | HADS-A | Decrease in HADS-A and STAI-state+trait at all subacute timepoints after psilocybin but not placebo | -/± | ^1^Data available only for 2 weeks follow-up |  |
|  |  |  |  |  |  |  |  |  |  |  | Spielberger’s Trait-Anxiety Inventory (state + trait version) | STAI |  |  |  |  |
|  |  |  |  |  |  |  |  |  |  |  | Death Anxiety Scale^1^ | DAS | No change in DAS |  |  |  |
| Carhart-Harris et al. | (2018) | B | Psilocybin | 2 sessions: 10 mg and 25 mg, 1 week apart (oral) | Patients with treatment resistant MDD | 20 | 44 (27-64) | 6 (30) | none | 1 week after 2nd session | Spielberger’s State-Trait Anxiety Inventory (trait version)^1^ | STAI | Decrease | - | ^1^Data available only for 1 week follow-up |  |
|  |  |  |  |  |  |  |  |  |  |  |  |  |  |  |  |  |
|  |  |  |  |  |  |  |  |  |  |  |  |  |  |  |  |  |
| Schmid & Liechti | (2018) | A | LSD | 200 µg (oral) | Healthy volunteers | 16 | 29 (25-51) | 8 (50) | crossover with placebo | 1 month | Spielberger’s State-Trait Anxiety Inventory (trait version) | STAI | No change | ± |  |  |
| Uthaug et al. | (2018) | C | Ayahuasca | Quantity n/a, per 200 mL: 189.4-915.4 mg DMT, 485.5-1261.7 mg harmine, and 38.1-892.0 mg harmaline (oral) | Ceremony-participants | 57 | n/a | 36 (63) | none | 1 day, 4 weeks | Depression, Anxiety, and Stress Scale-21 (Anxiety) | DASS-21 | No change | ± |  |  |
| Uthaug et al. | (2019) | C | 5-MeO-DMT | n/a (inhalation) | Participants of recreational 5-MeO-DMT retreats | 24 | 38 (SEM = 0.8)^1^ | 17 (40)^1^ | none | 1 day,1 month | Depression, Anxiety, and Stress Scale 21 (Anxiety) | DASS-21 | No change at 1 day Decrease at 4 weeks | -/± | ^1^Data of baseline sample n = 42 |  |
| Anderson et al. | (2020) | B | Psilocybin | 22-32 mg (oral) | demoralized older self-identified gay AIDS survivor men | 18 | 59.2 (4.4) | 0 (0) | none | 3 weeks | State-Trait Anxiety Inventory (state + trait version) | STAI | No change in STAI state, decrease in STAI trait | ±/- |  |  |
| Barrett et al. | (2020) | B | Psilocybin | 25 mg/70 kg (oral) | Healthy volunteers | 12 | 32 (7.5) | 7 (58) | none | 1 week, 1 month | Spielberger’s State-Trait Anxiety Inventory (state + trait version) | STAI | Decrease in state anxiety at 1 week, not at 1 month Significant decrease in trait anxiety at 1 month, not at 1 week | -/± |  |  |
| Uthaug et al. | (2020) | C | 5-MeO-DMT | Single dose 3-24 mg, summed total dose 17-61 mg (inhalation) | Participants of 5-MeO-DMT one-on-one-sessions | 11 | 33 (8.59) | 3 (27) | none | 1 week | Depression, Anxiety, and Stress Scale-21 (Anxiety) | DASS-21 | Decrease | - |  |  |
| Davis et al. | (2021) | A | Psilocybin | 2 sessions, 20mg/70kg and 30mg/70kg, 1.6 weeks apart (oral) | Patients with moderate to severe MDD | 13 | 43.6 (13.0) | 9 (69) | between group: waiting list | 4 weeks | Spielberger’s State-Trait Anxiety Inventory (state + trait version)^1^ | STAI | Greater reduction in all anxiety scores in the immediate treatment condition compared with waiting control group | - | ^1^Only available for week 4 |  |
|  |  |  |  |  |  |  |  |  |  |  | Hamilton Anxiety Scale^1^ | HAM-A |  |  |  |  |
| Dos Santos et al. | (2021) | A | Ayahuasca | 2 mL/kg, mean 0.68 mg/mL DMT, 0.52 mg/mL harmine, 0.62 mg/mL THH, 0.14 mg/mL harmaline | Volunteers with social anxiety disorder | 9 | 24.9 (19-32) | 15 (88)^1^ | between group: placebo | 7 days 14 days 21 days | Becks Anxiety Inventory | BAI | No change | ± | ^1^Of total sample (including control group) |  |
| Uthaug et al. | (2021) | C | Ayahuasca | 7-10 capsules 552mg ± 66.3 with of 3.6 ± 0.2 mg/g DMT, 10.1 mg/g ± 0.8 harmine, 0.7 ± 0.1 harmaline (mean ± SD) | Students of "ayahuasca school" | 14 | 40.2 (10.1)^1^ | 18 (60)^1^ | Between group: Placebo | 1 day | Depression, Anxiety, and Stress Scale 21 (Anxiety) | DASS-21 | Decrease in anxiety pre to post in ayahuasca and placebo group | ± | ^1^Data of total sample (including control group) |  |
| **Substance abuse** | | | | | | | | | | | | | | | |  |
| Johnson et al. | (2014) | B | Psilocybin | 2-3 sessions: 20 mg/70 kg and/or 30 mg/70 kg, 2 and 5 weeks apart (oral) | Nicotine-dependent smokers, otherwise psychiatrically healthy | 10 | 51 (26-65) | 5 (33) | none | 1 week, smoking cessation also assessed at weeks 2,3 after each dosing session | Questionnaire on Smoking Urges | QSU | Increased confidence to abstain | -/±/+ |  |  |
|  |  |  |  |  |  |  |  |  |  |  | Smoking Abstinence Self-Efficacy scale | SASE | Decreased craving and temptation to smoke |  |  |  |
|  |  |  |  |  |  |  |  |  |  |  | Wisconsin Smoking Withdrawal Scale | WSWS | Withdrawal peaked at 1 week and then decreased |  |  |  |
| Bogenschutz et al- | (2015) | B | Psilocybin | 2 sessions: 0.3 and 0.4 mg/kg, 4 weeks apart (oral) | Patients with active alcohol-dependency | 10 | 40 (25-56) | 4 (40) | none | 1 week,  4 weeks | Time-Line Follow Back Drinking days | TLFB | Decreased drinking days | -/± |  |  |
|  |  |  |  |  |  |  |  |  |  |  | Stages of Change Readiness and Treatment Eagerness Scale | SOCRATES 8A | No change in ambivalence and recognition Increase in taking steps |  |  |  |
|  |  |  |  |  |  |  |  |  |  |  | Alcohol Abstinence Self-Efficacy Scale | AASE | 1 week: increased confidence after 1^st^ and 2^nd^ dosing, decreased temptation only after 2^nd^ dosing 4 week: no change |  |  |  |
|  |  |  |  |  |  |  |  |  |  |  | Penn Alcohol Craving Scale | PACS | 1week: Decreased craving after 2^nd^ dosing 4 week: Decreased craving after both dosing sessions |  |  |  |
| **Psychosis** | | | | | | | | | | | | | | | |  |
| Gouzoulis-Mayfrank | (1999) | B | Psilocybin | 0.2mg/kg but not more than 15mg total dose (oral) | Healthy volunteers: physicians or psychologists with scientific or clinical interest in study | 8 | 31 (29-37) | 5 (63) | between group: 0.2 mg/kg d-amphetamine, or 2 mg/kg MDE, or placebo | 1 day,  7 days | Positive and Negative Symptom Scale | PANSS | No change | ± |  |  |
| Carhart-Harris et al. | (2016) | B | LSD | 75 µg (intravenous) | Healthy volunteers with prior experience with psychedelics | 20 | 31 (22-47) | 4 (20) | crossover with 10ml saline | 2 weeks | Peter’s Delusions Inventory (delusional ideation) | PDI | No change | ± |  |  |
|  |  |  |  |  |  |  |  |  |  |  |  |  |  |  |  |  |
|  |  |  |  |  |  |  |  |  |  |  |  |  |  |  |  |  |
| Jiménez-Garrido et al. | (2020) | C | Ayahuasca | n/a (oral) | Ceremony-participants, ayahuasca-naïve | 28 | 35 (20-65)^1^ | 28 (70)^1^ | none | 1 month | Community Assessment of Psychic Experience | CAPE | No change | ± | ^1^Data of baseline sample n = 40 (only study 1) |  |
| **Obsessive-compulsive symptoms** | | | | | | | | | | | | | | | |  |
| Moreno | (2006) | B | Psilocybin | Up to 4 sessions: 25, 100, 200, and 300 µg/kg, each at least 1 week apart (oral) | Patients with obsessive-compulsive disorder | 9 | 41 (26-62) | 2 (22) | none | 1 day after each session | Yale-Brown Obsessive Compulsive Scale | YBOCS | Decrease | - |  |  |
| **PTSD Symptoms** | | | | | | | | | | | | | | | |  |
| Anderson et al. | (2020) | B | Psilocybin | 22-32 mg (oral) | Demoralized older self-identified gay AIDS survivor men | 18 | 59.2 (4.4) | 0 (0) | none | 3 weeks | PTSD Checklist Scale-5 | PCL-5 | Decrease | - |  |  |
| **Personality/Values/Attitudes** | | | | | | | | | | | | | | | |  |
| Denber & West | (1958) | B | Mescaline sulfate | 0.5 g (injection) | Random selection of psychiatric patients | 9 | n/a | 4 (44) | none | 1 day | Minnesota Multiphasic Personality Inventory | MMPI | No change | ± |  |  |
| Ramsay et al. | (1963) | B | LSD | n/a | Hospitalized patients with alcohol addiction | 47 | 38 (n/a) | 4 (9) | none | 1-2 days | Allport-Vernon study of values |  | Increases in religious scale but not in other scales (theoretical, economic, esthetic, social, political) | +/± |  |  |
| Bottrill | (1969) | B | LSD | 400 µg (oral) | Volunteers, undergraduate students | 8 | 24 (4.58)^1^ | 0 (0) | between group: no drug | 1 week | Minnesota Multiphasic Personality Inventory | MMPI | Decrease in hypochondriasis, no change in other subscales | -/± | ^1^Data of baseline sample size n = 11 |  |
| Ludwig et al. | (1969) | B | LSD | 3 µg/kg (oral) | Patients with alcohol addiction | 132 ^1^ | n/a (21-55) | 0 (0) | between group: no drug | 10-14 days,  4 weeks | California Psychological Inventory | CPI | Changes in the direction of health or improvement in most measures, but not specific to LSD, also in controls | ± | ^1^Participants were allocated to one of three experimental groups: 1) LSD + hypnosis + psychotherapy, 2) LSD + psychotherapy 3) LSD |  |
| Kurland et al. | (1971) | A | LSD | 450 µg (oral) | Patients with alcohol addiction | 90 | n/a | n/a | between group: 50 µg LSD | 1 week | Minnesota Multiphasic Personality Inventory | MMPI | No change in comparison to control group. | ± |  |  |
|  |  |  |  |  |  |  |  |  |  |  | Eysenck Personality Inventory | EPI |  |  |  |  |
|  |  |  |  |  |  |  |  |  |  |  | Personal Orientation Inventory | POI |  |  |  |  |
|  |  |  |  |  |  |  |  |  |  |  | Psychiatric Evaluation Profile | PEP |  |  |  |  |
| Griffiths et al. | (2011) | A | Psilocybin | 4 sessions: 5, 10, 20, 30 mg/70 kg, in ascending or descending order, each one month apart (oral) | Healthy volunteers, Hallucinogen-naïve | 18 | 46 (29-62) | 10 (56) | crossover with placebo | 3-4 weeks after each session | Persisting Effects Questionnaire (attitudes towards life and self) | PEQ | Increasing effects as a function of dose in positive subscales. Negative ratings did not differ across the doses except for negative attitudes about self that showed increases at the two lowest doses. | +/- |  |  |
| Johnson et al. | (2014) | B | Psilocybin | 2-3 sessions: 20 mg/70 kg and/or 30 mg/70 kg, 2 and 5 weeks apart (oral) | Nicotine-dependent smokers, otherwise psychiatrically healthy | 10 | 51 (26-65) | 5 (33) | none | 1 week | Persisting Effects Questionnaire (attitudes towards life and self) | PEQ | Greater positive than negative effects across all subscales | + |  |  |
| Carhart-Harris et al. | (2016) | B | LSD | 75 µg (intravenous) | Healthy volunteers with prior experience with psychedelics | 20 | 31 (22-47) | 4 (20) | crossover with 10ml saline | 2 weeks | Revised NEO Personality Inventory | NEO-PI-R | Increased openness after LSD but not placebo, no change in other subscales | +/± |  |  |
|  |  |  |  |  |  |  |  |  |  |  | Revised Life Orientation Test | LOT-R | Increased optimism |  |  |  |
| Ross et al. | (2016) | A | Psilocybin | 0.3 mg/kg (oral) | Patients with cancer-related anxiety and depression | 29 | 56 (22-75) | 18 (62) | crossover with 250mg niacin | 2 weeks | Persisting Effects Questionnaire (attitudes towards life and self) | PEQ | Increases in positive subscales. No changes in negative subscales. | + |  |  |
| Lyons & Carhart-Harris^1^ | (2018a) | B | Psilocybin | 2 sessions: 10 mg and 25 mg, 1 week apart (oral) | Patients with treatment resistant major depressive disorder | 7 | 48 (SEM = 4.5) | 0 (0) | between group: healthy controls, no drug | 1 week | Political Perspectives Questionnaire | PPQ-5 | Decrease in authoritarianism political perspective after psilocybin but not placebo | -/+ | ^1^Two references are counted as one study, as both stem from the same sample (Carhart-Harris et al., 2018) |  |
|  |  |  |  |  |  |  |  |  |  |  | Nature Relatedness Scale | NR-6 | Increase in nature relatedness after psilocybin but not placebo |  |  |  |
| Lyons & Carhart-Harris^1^ | (2018b) | B | Psilocybin | 2 sessions: 10 mg and 25 mg, 1 week apart (oral) | Patients with treatment resistant major depressive disorder | 15 | 45 (SEM = 2.9) | 4 (27) | between group: healthy controls, no drug | 1 week | Prediction Of Future Life Events task (cognitive biases in depression) | POFLE | After treatment significantly higher probability estimates for desirable than undesirable events and higher accuracy in patients, alignment to behavior of healthy controls. Significantly reduced pessimism in patients, alleviated to control levels |  |  |  |
|  |  |  |  |  |  |  |  |  |  |  |  |  |  |  |  |  |
|  |  |  |  |  |  |  |  |  |  |  |  |  |  |  |  |  |
| Schmid & Liechti | (2018) | A | LSD | 200 µg (oral) | Healthy volunteers | 16 | 29 (25-51) | 8 (50) | crossover with placebo | 1 month | Neuroticism-Extraversion-Openness Five-Factors-Inventory | NEO-FFI | No change | ±/+ |  |  |
|  |  |  |  |  |  |  |  |  |  |  | Persisting Effects Questionnaire (attitudes towards life and self) | PEQ | Increases in positive subscales. No changes in negative subscales. Compared with assumption of no change (rating = 0). |  |  |  |
| Barrett et al. | (2020) | B | Psilocybin | 25 mg/70 kg (oral) | Healthy volunteers | 12 | 32 (7.5) | 7 (58) | none | 1 week, 1 month | Big Five Inventory^1^ | BFI | Increase in conscientiousness, no change in other subscales | +/± | ^1^Data available only for 1 month follow-up |  |
|  |  |  |  |  |  |  |  |  |  |  | Tellegen Absorption Scale^1^ | TAS | Increased absorption |  |  |  |
|  |  |  |  |  |  |  |  |  |  |  | Dispositional Positive Emotions Scale | DPES | Increases in all subscales (joy, content, pride, compassion, amusement) at both time points |  |  |  |
| Jiménez-Garrido et al. | (2020) | C | Ayahuasca | n/a (oral) | Ceremony-participants, ayahuasca-naïve | 28 | 35 (20-65)^1^ | 28 (70)^1^ | none | 1 month | Temperament and Character Inventory-Revised-67 | TCI-R-67 | No change | ± | ^1^ Data of baseline sample n = 40 (only study 1) |  |
| Netzband et al. | (2020) | C | Ayahuasca | 6 sessions in 12 days: n/a (oral) | Attendees of an ayahuasca ceremonies program | 24 | 37.6 | 9 (38) | between group: Tourists with no experience | 1 day after last session | NEO Personality Inventory - 3 | NEO-PI3 | Decresase in neuroticism, increase in agreeableness, no change in other dimensions | +/-/± |  |  |
| Mans et al. | (2021) | C | Mixed | n/a | Volunteers participating in online survey | 212-315 | 28.9 (10.45) | 165 (25)^1^ | none | 2 weeks,  4 weeks | Revised Life Orientation Test | LOT-R | Increase | + | ^1^Data from baseline sample size n = 654. Data are (sub-)sample of Haijen et al., 2018, included because new data are presented |  |
|  |  |  |  |  |  |  |  |  |  |  | Rosenberg Self-Esteem Scale | RSE | Increase |  |  |  |
|  |  |  |  |  |  |  |  |  |  |  | Ten-item Personality Inventory -Emotional Stability | TIPI-ES | Increase |  |  |  |
|  |  |  |  |  |  |  |  |  |  |  | Brief Resilience Scale | BRS | Increase |  |  |  |
|  |  |  |  |  |  |  |  |  |  |  | Meaning in Life Questionnaire (Presence) | MLQ-P | Increase |  |  |  |
|  |  |  |  |  |  |  |  |  |  |  | Gratitude Questionnaire | GQ-6 | Increase |  |  |  |
| **Mysticism/Spirituality** | | | | | | | | | | | | | | | |  |
| Trichter et al. | (2009) | C | Ayahuasca | n/a | Ceremony-participants, ayahuasca-naïve | 49 | 33 (n/a) | 26 (53) | between group: no drug | 1 week, 4 weeks | Mysticism Scale | MS | No change | ± |  |  |
| Griffiths et al. | (2011) | A | Psilocybin | 4 sessions: 5, 10, 20, 30 mg/70 kg, in ascending or descending order, each one month apart (oral) | Healthy volunteers, Hallucinogen-naïve | 18 | 46 (29-62) | 10 (56) | crossover with placebo | 3-4 weeks after each session | Persisting Effects Questionnaire (Spirituality) | PEQ | Increasing effects as a function of dose in positive subscales. Negative ratings did not differ. | + |  |  |
| Johnson et al. | (2014) | B | Psilocybin | 2-3 sessions: 20 mg/70 kg and/or 30 mg/70 kg, 2 and 5 weeks apart (oral) | Nicotine-dependent smokers, otherwise psychiatrically healthy | 10 | 51 (26-65) | 5 (33) | none | 1 week | Mysticism Scale^1^ | MS | Increase | + | ^1^data available only 1 week after 2nd and 3^rd^ psilocybin session |  |
|  |  |  |  |  |  |  |  |  |  |  | Persisting Effects Questionnaire (Spirituality) | PEQ | Greater positive than negative effects. |  |  |  |
| Ross et al. | (2016) | A | Psilocybin | 0.3 mg/kg (oral) | Patients with cancer-related anxiety and depression | 29 | 56 (22-75) | 18 (62) | crossover with 250mg niacin | 2 weeks | Death Transcendence Scale | DTS | No change | ±/+ |  |  |
|  |  |  |  |  |  |  |  |  |  |  | Persisting Effects Questionnaire (Spirituality) | PEQ | Increases in positive subscale. No changes in negative subscale. |  |  |  |
| Schmid & Liechti | (2018) | A | LSD | 200 µg (oral) | Healthy volunteers | 16 | 29 (25-51) | 8 (50) | crossover with placebo^1^ | 1 month | Mysticism Scale | MS | Increases in introvertive and extrovertive mysticism, and in the total score. No change in interpretation | ±/+ | ^1^subacute effects were performed with a within-group pre/post analysis |  |
|  |  |  |  |  |  |  |  |  |  |  | Death Transcendence Scale | DTS | Increases in mysticism and total score. No change in the other subscales: religious, nature, creative, biosocial |  |  |  |
| Mans et al. | (2021) | C | Mixed | n/a | Volunteers participating in online survey | 212-315 | 28.9 (10.45) | 165 (25)^1^ | none | 2 weeks,  4 weeks | Spiritual Transcendence Scale (Universality) | STS-U | No change | ± | ^1^data from baseline sample size n = 654. Data are (sub-)sample of Haijen et al., 2018, included because new data are presented |  |
|  |  |  |  |  |  |  |  |  |  |  | Spiritual and Religious Attitudes in Dealing with Illness—modified short form (Trust) | SpREUK-SF-T | No change |  |  |  |
|  | | | | | | | | | | | | | | | |  |
|  | | | | | | | | | | | | | | | |  |
| **Creativity/Flexibility** | | | | | | | | | | | | | | | |  |
| McGlothlin et al. | (1964) | B | LSD | 200 µg (oral) | Most were employees of a think tank | 15 | 36 (26-49) | 5 (33) | between group: no drug | 1 week | Guilfords’ divergent thinking battery |  | No change | ± |  |  |
|  |  |  |  |  |  |  |  |  |  |  | Mednick’s Remote Associations |  |  |  |  |  |
| Frecska et al. | (2012) | C | Ayahuasca | 4-5 sessions over a period of 2 weeks: total of 583 ± 315.8 mL (mean ± SD), 0.73 mg/mL DMT, 1.36 mg/ml harmine, 1.05 mg/mL, tetrahydroharmine (oral) | Ceremony-participants | 40 | 31 (7.7) | 23 (58) | between group: no drug | 2 days after the 2 weeks | Torrance Tests of Creative Thinking | TTCT | No changes on creativity measures such as fluency, relative flexibility, and relative originality. Increase in the number of highly original solutions both in blank circle use and figure completion. Ambigous results concerning phosphenic responses | +/± |  |  |
| Uthaug et al. | (2018) | C | Ayahuasca | Quantity n/a, per 200 mL: 189.4-915.4 mg DMT, 485.5-1261.7 mg harmine, and 38.1-892.0 mg harmaline (oral) | Ceremony-participants | 57 | n/a | 36 (63) | none | 1 day, 4 weeks | Picture Concept Task | PCT | Increase in convergent thinking not at 1 day, but at 1 month. No changes in divergent thinking. | -/± |  |  |
| Mason et al. | (2019) | C | Psilocybin | Mean 27.1 mg psilocin (oral) | Participants of psilocybin retreats | 22-50 | 35 (8.9)^1^ | 26 (47)^1^ | none | 1 day, 7 days | Picture Concept Task | PCT | Increase in convergent thinking not at 1 day, but at 1 week. Increase in divergent thinking (fluency and originality) at 1 day, but not at 1 week. No change in the ratio of originality/fluency. | -/+/± | ^1^Of the baseline sample of n = 55 |  |
| Uthaug et al. | (2019) | C | 5-MeO-DMT | Dose n/a (inhalation) | Participants of recreational 5-MeO-DMT retreats | 24 | 38 (SEM = 0.8)^1^ | 17 (40)^1^ | none | 1 day, 1 month | Picture Concept Task | PCT | Increase in convergent thinking at both time points. No change in divergent thinking | -/± | ^1^Data of baseline sample n = 42 |  |
| Murphy-Beiner & Soar | (2020) | C | Ayahuasca | n/a (oral) | Healthy ceremony-participants | 48 | 38 (7.21) | 26 (54) | none | 1 day | Cognitive Flexibility Scale | CFS | Increase | +/± |  |  |
|  |  |  |  |  |  |  |  |  |  |  | Wisconsin Picture Card Sorting Task | WCST | No change in RT but increase in % of correct responses |  |  |  |
|  |  |  |  |  |  |  |  |  |  |  | Stroop colour and word task | Stroop | No effect in interference but reduced errors in incongruent condition, no change in reaction times |  |  |  |
| Mason et al. | (2021) | A | Psilocybin | 0.17 mg/kg | Healthy volunteers, with prior experience with psychedelics (not last 3 month) | 30 | 22.73 (2.90) | 12 (40) | between group: placebo | 7 days | Picture Concept Task | PCT | Decreased convergent thinking compared to placebo, no change in fluency and originality | +/± |  |  |
|  |  |  |  |  |  |  |  |  |  |  | Alternative Uses Task | AUT | Increased scores of novelty compared to placebo, no change in fluency and originality |  |  |  |
| **Mindfulness/Acceptance/Emotion regulation** | | | | | | | | | | | | | | | |  |
| Soler et al. | (2016) | B | Ayahuasca | Quantity n/a, on average 43.6 mg DMT, range 28.8–69.8 (oral) | Healthy volunteers, most of them with prior ayahuasca experience | 25 | 44 (12) | 14 (56) | none | 1 day | Five Facet Mindfulness Questionnaire | FFMQ | Increases in non-judging and non-reacting.No change in observing, describing, acting | +/± |  |  |
|  |  |  |  |  |  |  |  |  |  |  | Experience Questionnaire | EQ | Increase |  |  |  |
| Sampedro et al. | (2017) | B | Ayahuasca | 148 ± 29 mL with 45 ± 9 mg DMT, 126 ± 25 mg harmine, 26 ± 5 mg tetrahydro­harmine, and 5 ± 1 mg harmaline (mean ± SD) (oral) | Healthy volunteers with prior ayahuasca experience | 16 | 39 (7.8) | 6 (38) | none | 1 day | Five Facet Mindfulness Questionnaire | FFMQ | Increases in non-judging and non-reacting. No change in observing, describing, acting. | +/± |  |  |
|  |  |  |  |  |  |  |  |  |  |  | Experience Questionnaire | EQ | Increase |  |  |  |
|  |  |  |  |  |  |  |  |  |  |  | Short version of the Self-Compassion questionnaire | SC | Increase |  |  |  |
| Soler et al. | (2018) | B | Ayahuasca | 4 sessions, quantity and DMT n/a, each 1 week apart (oral) | Volunteers | 10 | 50 (14.7) | 7 (70) | between group: participants of MBSR course | 1 day after the last session | Five Facet Mindfulness Questionnaire | FFMQ | Increase in non-judging. No change in observing, describing, acting, and non-reacting. However, changes were greater in control group. | ± |  |  |
|  |  |  |  |  |  |  |  |  |  |  | Experience Questionnaire | EQ | No change |  |  |  |
|  |  |  |  |  |  |  |  |  |  |  |  |  |  |  |  |  |
|  |  |  |  |  |  |  |  |  |  |  |  |  |  |  |  |  |
| Uthaug et al. | (2018) | C | Ayahuasca | Quantity n/a, per 200 mL: 189.4-915.4 mg DMT, 485.5-1261.7 mg harmine, and 38.1-892.0 mg harmaline (oral) | Ceremony-participants | 57 | n/a | 36 (63) | none | 1 day, 4 weeks | Five Facets Mindfulness Questionnaire-39 | FFMQ | Increases in observing, acting, non-judging at 1 day, but not at 1 month. No change in describing, and non-reaction. | +/± |  |  |
| Domínguez-Clavé et al. | (2019) | C | Ayahuasca | n/a (oral) | Ceremony participants | 45 | 39.89 (7.48) | 27 (60) | none | 1 day | Difficulties in Emotion Regulation | DERS | Decreases in emotional non-acceptance, emotional interference and lack of control. No change in emotional awareness, lack of clarity. | +/± |  |  |
|  |  |  |  |  |  |  |  |  |  |  | Five Facet Mindfulness Questionnaire - short form | FFMQ-SF | Increases in observing, awareness, non-judging, and non-reacting. No change in describing. |  |  |  |
|  |  |  |  |  |  |  |  |  |  |  | Experiences Questionnaire | EQ | Increase |  |  |  |
| Uthaug et al. | (2019) | C | 5-MeO-DMT | n/a (inhalation) | Participants of recreational 5-MeO-DMT retreats | 24 | 38 (SEM = 0.8)^1^ | 17 (40)^1^ | none | 1 day, 1 month | Five Facets of Mindfulness Questionnaire-15 | FFMQ-15 | Increases in non-judging, acting not at 1 day, but at 1 month. No significant changes in observing, describing and non-reacting. | +/± | ^1^Data of baseline sample n = 42 |  |
| Jiménez-Garrido et al. | (2020) | C | Ayahuasca | n/a (oral) | Ceremony-participants, ayahuasca-naïve | 28 | 35 (20-65)^1^ | 28 (70)^1^ | none | 1 month | Acceptance and Action Questionnaire | AAQ-II | No change | ± | ^1^Data of baseline sample n = 40 (study 1) |  |
| Murphy-Beiner & Soar | (2020) | C | Ayahuasca | n/a (oral) | Healthy ceremony-participants | 48 | 38 (7.21) | 26 (54) | none | 1 day | Five Facets Mindfulness Questionnaire-Short Form | FFMQ-24 | Increase in total score and all subscales except in non-judging | +/± |  |  |
|  |  |  |  |  |  |  |  |  |  |  | Experiences Questionnaire | EQ | Increase |  |  |  |
| Uthaug et al. | (2020) | C | 5-MeO-DMT | Single dose 3-24 mg, summed total dose 17-61 mg (inhalation) | Participants of 5-MeO-DMT one-on-one-sessions | 11 | 33 (8.59) | 3 (27) | none | 1 week | Five Facets Mindfulness Questionnaire-39 | FFMQ-39 | Increase in non-judgement at both time points. No changes in other subscales. | +/± |  |  |
| Zeifman et al. | (2020) | C | Mixed | n/a | Volunteers in online survey intending to take a psychedelic | 104 | 29.28 (9.94) | 31 (30) | none | 2 weeks,  4 weeks | Brief Experiential Avoidance Questionnaire | BEAQ | Reductions in avoidance at both follow-ups | + |  |  |
|  |  |  |  |  | Volunteers in online survey intending to attended psychedelic ceremony | 254 | 43.61 (12.46) | 115 (45) | none | 4 weeks | Brief Experiential Avoidance Questionnaire | BEAQ | Reductions in avoidance | + |  |  |
| Mans et al. | (2021) | C | Mixed | n/a | Volunteers participating in online survey | 212-315 | 28.9 (10.45) | 165 (25)^1^ | none | 2 weeks,  4 weeks | Revised Cognitive and Affective Mindfulness Scale | CAMS-R | Increase | + | ^1^Data from baseline sample size n = 654. Data are (sub-)sample of Haijen et al., 2018, included because new data are presented |  |
|  |  |  |  |  |  |  |  |  |  |  | Acceptance and Action Questionnaire-II | AAQ-II | Decrease |  |  |  |
| Uthaug et al. | (2021) | C | Ayahuasca | 7-10 capsules 552mg ± 66.3 with of 3.6 ± 0.2 mg/g DMT, 10.1 mg/g ± 0.8 harmine, 0.7 ± 0.1 harmaline (mean ± SD) | Students of "ayahuasca school" | 14 | 40.2 (10.1)^1^ | 18 (60)^1^ | Between group: Placebo | 1 day | Five Facet Mindfulness Questionnaire | FFMQ | No change | ± | ^1^Data of total sample (including control group) |  |
| Wießner et al. | (2021) | A | LSD | 50 µg (oral) | Healthy volunteers | 24 | 35 (11) | 8 (33) | Crossover: Placebo (alcohol solution) | 1 day,  2 weeks | Five Facet Mindfulness Questionnaire | FFMQ | No change | ± |  |  |
|  |  |  |  |  |  |  |  |  |  |  | Mindful Attention Awareness Scale | MAAS | No change |  |  |  |
|  |  |  |  |  |  |  |  |  |  |  | Experiences Questionnaire | EQ | No change |  |  |  |
| **Social Effects/Empathy/Compassion** | | | | | | | | | | | | | | | |  |
| McGlothlin et al. | (1964) | B | LSD | 200 µg (oral) | Most were employees of a think tank | 15 | 36 (26-49) | 5 (33) | between group: no drug | 1 week | Marlowe-Crowne Social Desirability Scale (defensiveness) |  | No change | ± |  |  |
| Griffiths et al. | (2011) | A | Psilocybin | 4 sessions: 5, 10, 20, 30 mg/70 kg, in ascending or descending order, each one month apart (oral) | Healthy volunteers, Hallucinogen-naïve | 18 | 46 (29-62) | 10 (56) | crossover with placebo | 3-4 weeks after each session | Persisting Effects Questionnaire (Social effects) | PEQ | Increasing effects as a function of dose in positive subscales. Negative ratings did not differ across the doses. | + |  |  |
| Johnson et al. | (2014) | B | Psilocybin | 2-3 sessions: 20 mg/70 kg and/or 30 mg/70 kg, 2 and 5 weeks apart (oral) | Nicotine-dependent smokers, otherwise psychiatrically healthy | 10 | 51 (26-65) | 5 (33) | none | 1 week | Persisting Effects Questionnaire (Social effects) | PEQ | Greater positive than negative effects | + |  |  |
| Ross et al. | (2016) | A | Psilocybin | 0.3 mg/kg (oral) | Patients with cancer-related anxiety and depression | 29 | 56 (22-75) | 18 (62) | crossover with 250mg niacin | 2 weeks | Persisting Effects Questionnaire (Social effects) | PEQ | Increases in positive, no change in negative effects. | + |  |  |
| Schmid & Liechti | (2018) | A | LSD | 200 µg (oral) | Healthy volunteers | 16 | 29 (25-51) | 8 (50) | crossover with placebo | 1 month | Persisting Effects Questionnaire (Social effects) | PEQ | Increases in positive, no change in negative effects. Compared with assumption of no change (rating = 0). | + |  |  |
|  |  |  |  |  |  |  |  |  |  |  |  |  |  |  |  |  |
|  |  |  |  |  |  |  |  |  |  |  |  |  |  |  |  |  |
| Stroud et al. | (2018) | B | Psilocybin | 2 sessions: 10 mg and 25 mg, 1 week apart (oral) | Patients with treatment resistant major depressive disorder | 17 | 45 (11.5) | 6 (35) | between group: healthy controls, no drug | 1 week after last session | Dynamic Emotional Expression Recognition Task | DEER-T | Significant group x time interaction on speed of emotion recognition but not on accuracy, discrimination, or response bias. At baseline, patients were slower at recognizing facial emotions compared with controls. After psilocybin, this difference was remediated. | +/± |  |  |
| Mason et al. | (2019) | C | Psilocybin | Mean 27.1 mg psilocin (oral) | Participants of psilocybin retreats | 22-50 | 35 (8.9)^1^ | 26 (47)^1^ | none | 1 day, 7 days | Multifaceted empathy test | MET | Cognitive empathy: no change. Emotional empathy (EE): Explicit: Increases in average and negative at 1 day, but not at 1 week. No change in positive Implicit: Increases in average, positive, and negative at 1 day. At 7 day only significant increase in negative. | +/± | ^1^Of the baseline sample of n = 55 |  |
| Mans et al. | (2021) | C | Mixed | n/a | Volunteers participating in online survey | 212-315 | 28.9 (10.45) | 165 (25)^1^ | none | 2 weeks,  4 weeks | Santa Clara Brief Compassion Scale | SCBCS | No change | ±/+ | ^1^Data from baseline sample size n = 654. Data are (sub-)sample of Haijen et al., 2018, included because new data are presented |  |
|  |  |  |  |  |  |  |  |  |  |  | Social Connectedness Scale | SCS | Increase |  |  |  |
| Uthaug et al. | (2021) | C | Ayahuasca | 7-10 capsules 552mg ± 66.3 with of 3.6 ± 0.2 mg/g DMT, 10.1 mg/g ± 0.8 harmine, 0.7 ± 0.1 harmaline (mean ± SD) | Students of "ayahuasca school" | 14 | 40.2 (10.1)^1^ | 18 (60)^1^ | Between group: Placebo | 1 day | Multifaceted Empathy Test | MET | Increase in implicit empathy towards negative stimuli, no change in explicit emapthy | +/± | ^1^Data of total sample (including control group) |  |
| **Behavioral change** | | | | | | | | | | | | | | | |  |
| Griffiths et al. | (2011) | A | Psilocybin | 4 sessions: 5, 10, 20, 30 mg/70 kg, in ascending or descending order, each one month apart (oral) | Healthy volunteers, Hallucinogen-naïve | 18 | 46 (29-62) | 10 (56) | crossover with placebo | 3-4 weeks after each session | Persisting Effects Questionnaire (Behavioral Change) | PEQ | Increasing effects as a function of dose in positive subscales. Negative ratings did not differ. | + |  |  |
| Johnson et al. | (2014) | B | Psilocybin | 2-3 sessions: 20 mg/70 kg and/or 30 mg/70 kg, 2 and 5 weeks apart (oral) | Nicotine-dependent smokers, otherwise psychiatrically healthy | 10 | 51 (26-65) | 5 (33) | none | 1 week | Persisting Effects Questionnaire (Behavioral Change) | PEQ | Greater positive than negative effects. | + |  |  |
| Ross et al. | (2016) | A | Psilocybin | 0.3 mg/kg (oral) | Patients with cancer-related anxiety and depression | 29 | 56 (22-75) | 18 (62) | crossover with 250mg niacin | 2 weeks | Persisting Effects Questionnaire (Behavioral Change) | PEQ | Increases in positive subscale. No changes in negative subscale. | + |  |  |
| Schmid & Liechti | (2018) | A | LSD | 200 µg (oral) | Healthy volunteers | 16 | 29 (25-51) | 8 (50) | crossover with placebo | 1 month | Persisting Effects Questionnaire (Behavioral Change) | PEQ | Increases in positive subscale. No changes in negative subscale. Compared with assumption of no change (rating = 0). | + |  |  |
| **Cognitive Performance** | | | | | | | | | | | | | | | |  |
| Kurland et al. | (1971) | A | LSD | 450 µg (oral) | Patients with alcohol addiction | 90 | n/a | n/a | between group: 50 µg LSD | 1 week | Intelligence: Wechsler Adult Intelligence Scale | WAIS | No change | ± |  |  |
|  |  |  |  |  |  |  |  |  |  |  | Raven Progressive Matrices | RPM |  |  |  |  |
|  |  |  |  |  |  |  |  |  |  |  | Visual perception: Benton Visual Retention Test | BVRT |  |  |  |  |
|  |  |  |  |  |  |  |  |  |  |  | Embedded Figures Test | EFT |  |  |  |  |
| Anderson et al. | (2020) | B | Psilocybin | 22-32 mg (oral) | Demoralized older self-identified gay AIDS survivor men | 18 | 59.2 (4.4) | 0 (0) | none | 3 weeks | Montreal Cognitive Assessment | MoCA | No change | ± |  |  |
| **Complaints/Other drug effects** | | | | | | | | | | | | | | | |  |
| Gouzoulis-Mayfrank et al. | (1999) | B | Psilocybin | 0.2mg/kg but not more than 15mg total dose (oral) | Healthy volunteers: physicians or psychologists with scientific or clinical interest in study | 8 | 31 (29-37) | 5 (63) | between group: 0.2 mg/kg d-amphetamine, or 2 mg/kg MDE, or placebo | 1 day,  7 days | Vegetative Lability Scale B-L/Beschwerdeliste | LC/BL | Increase after 1 day, but not 1 week compared to baseline (within group comparison) | +/± |  |  |
| Hasler et al. | (2002) | B | Psilocybin | 212 ± 25 µg/kg (oral) | Healthy volunteers | 8 | 33 (27-46) | 4 (50) | none | 1 week | List of Complaints/Beschwerdeliste | LC/BL | No change | ± |  |  |
|  |  |  |  |  |  |  |  |  |  |  |  |  |  |  |  |  |
|  |  |  |  |  |  |  |  |  |  |  |  |  |  |  |  |  |
| Schmid et al. | (2015) | A | LSD | 200 µg (oral) | Healthy volunteers | 16 | 29 (25-51) | 8 (50) | crossover with placebo | 1 day, 3 days | List of Complaints/Beschwerdeliste | LC/BL | No change | ± |  |  |
|  |  |  |  |  |  |  |  |  |  |  | Addiction Research Center Inventory (subjective drug effects)^1^ | ARCI | No change |  | ^1^Only at day 1 |  |
| Dolder et al. | (2016) | A | LSD | 100 µg (oral) | Healthy volunteers, mostly hallucinogen-naive | 24^1^ | 33 (25-60) | 12 (50) | crossover with placebo | 1 day, 3 days | List of Complaints/Beschwerdeliste | LC/BL | No difference to placebo | ± | ^1^Only data from study 1 reported, data of study 2 already reported in Schmid et al. 2015 |  |
| Schindler et al. | (2021) | A | Psilocybin | 0.143 mg/kg | Migraine patients | 10 | 40.5 (SEM = 4.4) | 7 (70) | crossover with placebo | 2 weeks | Headache diary | Headache diary | Greater reduction in migraine frequency, weekly migraine attacks, pain severity, attack-related functional impairment, weekly migraine abortive days after psilocybin than placebo; The time to the first attack was statistically equivocal, but the time to the second attack was significantly greater after psilocybin; no differences in migraine attack duration or assoicated symptom ratings (photophobia, phonophobia, nausea/vomiting) | -/ ± |  |  |
| ^a^changes are only reported if they reached statistical significance | | | | | | | | | | | | | | | |  |
| ^b^the orientation of summary measures (+/-) always indicates increase or decrease in relation to the domain assessed. This does not necessarily coincide with the orientation of the scale, e.g., a high level of functioning measured in the GAF indicates a lower psychopathology. | | | | | | | | | | | | | | | |  |

| Table S2: Frequency and total sample size of studies that reported subacute effects by outcome domain | | | | | | |
| --- | --- | --- | --- | --- | --- | --- |
|  |  | **No** |  | **Direction of change** | | |
|  | **Total**  **(n)** | **Change**  **(n)** | **Change**  **(n)** | **Increase**  **(n)** | **Decrease**  **(n)** | **Other**  **(n)** |
| Wellbeing/Quality of Life/Satisfaction with life | 8 | 2 | 6 | 6 | 0 |  |
|  | (570) | (77) | (493) | (493) | (0) |  |
| Mood | 10 | 4 | 6 | 5 | 0 | 1 |
|  | (149) | (56) | (93) | (85) | (0) | (8) |
| General indicators of Psychopathology | 8 | 1 | 7 | 0 | 7 |  |
|  | (155) | (14) | (141) | (0) | (141) |  |
| Suicidality | 4 | 2 | 2 | 0 | 2 |  |
|  | (389) | (31) | (358) | (0) | (358) |  |
| Depression/Stress/Grief | 18 | 4 | 14 | 0 | 14 |  |
|  | (956) | (62) | (894) | (0) | (894) |  |
| Mania | 3 | 3 | 0 |  |  |  |
|  | (31) | (31) | (0) |  |  |  |
| Anxiety | 13 | 5 | 8 | 0 | 8 |  |
|  | (243) | (104) | (139) | (0) | (139) |  |
| Substance abuse | 2 | 0 | 2 | 0 | 2 |  |
|  | (20) | (0) | (20) | (0) | (20) |  |
| Psychosis | 3 | 3 | 0 |  |  |  |
|  | (56) | (56) | (0) |  |  |  |
| Obsessive-compulsive symptoms | 1 | 0 | 1 | 0 | 1 |  |
|  | (9) | (0) | (9) | (0) | (9) |  |
| PTSD symptoms | 1 | 0 | 1 | 0 | 1 |  |
|  | (18) | (0) | (18) | (0) | (18) |  |
| Personality/Values/Attitudes | 15 | 4 | 11 |  |  | 11 |
|  | (773) | (259) | (514) |  |  | (514) |
| Mysticism/Spirituality | 6 | 2 | 4 | 4 | 0 |  |
|  | (437) | (364) | (73) | (73) | (0) |  |
| Creativity/Flexibility | 7 | 1 | 6 | 3.5 | 2.5 |  |
|  | (264) | (15) | (249) | (143)^1^ | (106)^1^ |  |
| Mindfulness/Acceptance/Emotion regulation | 14 | 4 | 10 | 10 | 0 |  |
|  | (975) | (76) | (899) | (899) | (0) |  |
| Social Effects/Empathy/Compassion | 9 | 1 | 8 | 8 | 0 |  |
|  | (484) | (15) | (469) | (469) | (0) |  |
| Positive Behavioral Change | 4 | 0 | 4 | 4 | 0 |  |
|  | (73) | (0) | (73) | (73) | (0) |  |
| Cognitive Performance | 2 | 2 | 0 |  |  |  |
|  | (108) | (108) | (0) |  |  |  |
| Complaints/Other drug effects | 5 | 3 | 2 | 1 | 1 |  |
|  | (66) | (48) | (18) | (8) | (10) |  |
| ^1^ One study observed an increase in convergent and divergent thinking at different subacute assessment points and was therefore classified half as increase and half as decrease. | | | | | |  |

| 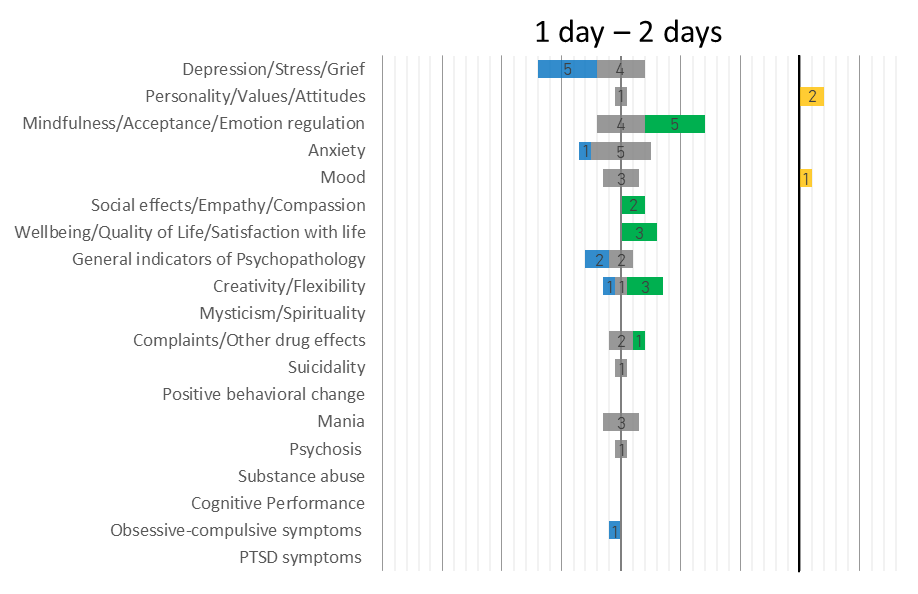 |
| --- |
| 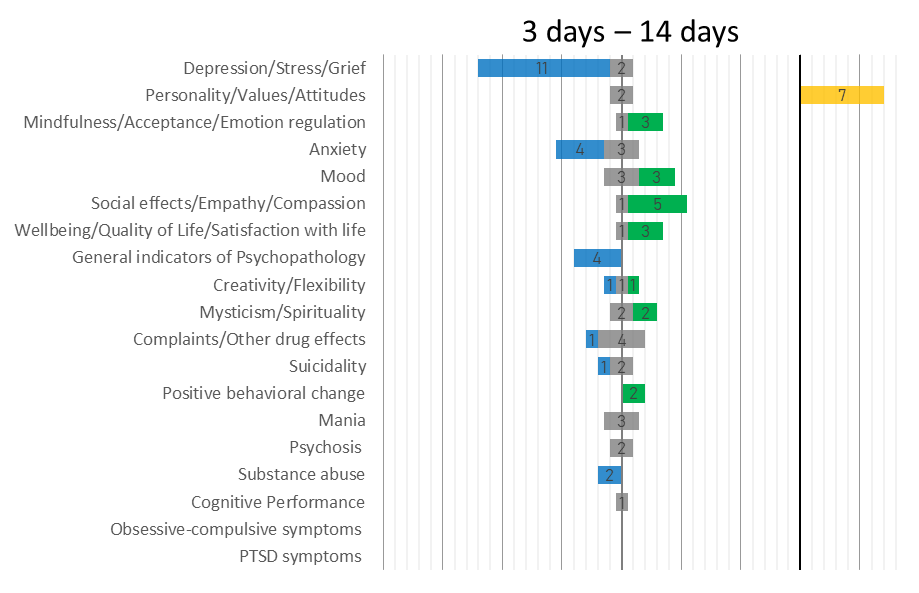 |
| 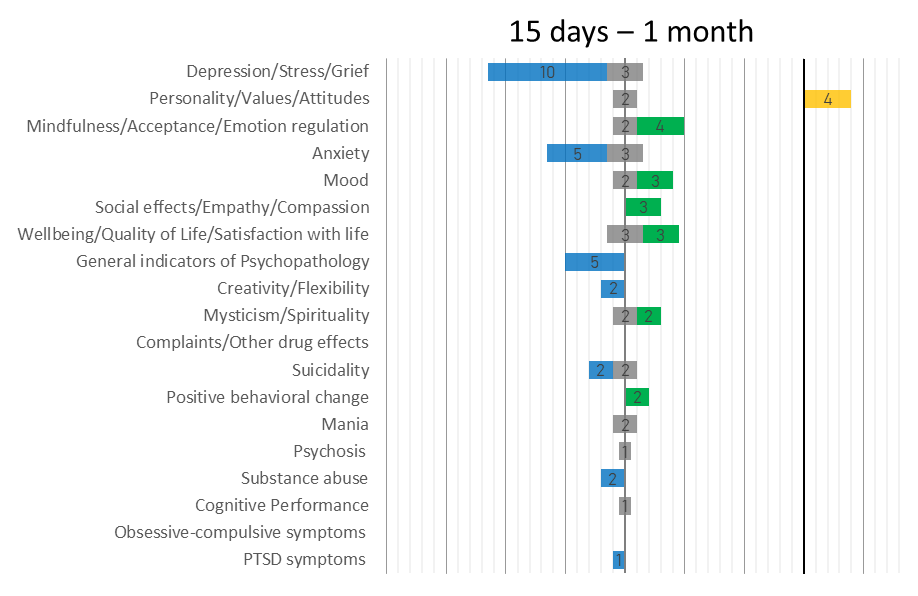 |
| 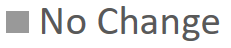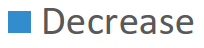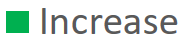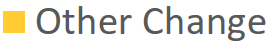 |
| Figure S2: Subacute effects across time |

| Table S4: Subacute adverse events of individual studies | | | | | |
| --- | --- | --- | --- | --- | --- |
| **Author** | **Year** | **Subacute adverse reactions** | | | **Reference** |
|  |  | **Not mentioned** | **None** | **Repor­ted** |  |
| Denber & West | 1958 |  |  | X | n = 1 psychiatric disturbance lasting a week  One patient “was so disturbed that he could not be tested until 7 days after the injection.” (p. 546) |
| Lebovits et al. | 1960 | X |  |  |  |
| Ramsay et al. | 1963 | X |  |  |  |
| McGlothlin et al. | 1964 | X |  |  |  |
| Bottrill | 1969 | X |  |  |  |
| Ludwig et al. | 1969 |  | X |  | “Virtually all patients seemed sufficiently prepared so as to experience the panoramic, spectacular effects of this drug without any adverse reactions (only two LSD sessions had to be terminated) - For the most part, especially with the hypnodelic and psychedelic conditions, the patients’ responses during therapy and their enthusiastic testimonials immediately following therapy were all that could have been hoped for. The difficulty was that these claims had little impact on the criterion measures for treatment outcome.” (p. 67) |
| Kurland et al. | 1971 |  |  | X | n = 1 adverse event, no specific description:  “Only one adverse reaction has been observed in our entire series, of well over 200 alcoholics treated with either high or low dose to date (June 1970). Furthermore, even in this one case, the reaction was reversed by conventional therapy.” (p. 91) |
| Gouzoulis-Mayfrank et al. | 1999 |  |  | X | Adverse effects only reported on group level: Significant increase of the B-L vegetative lability score at 1 days but not 7 days. (p. 45) |
| Hasler et al. | 2002 |  | X |  | “Only slight and short-term physical adverse reactions such as nausea and dizziness were observed during onset of PY effects in some subjects. None of our volunteers reported negative delayed effects in the follow-up Lists of Complaints.” p.335 |
| Hasler et al. | 2004 |  | X |  | “All PY-induced [psychological] symptoms were worn off completely 6–8 h after drug administration.” (p. 152) “Our investigations provided no cause for concern that administration of PY to healthy subjects is hazardous with respect to somatic health. However, as our data revealed tendencies of PY to temporarily increase blood pressure, we advise subjects suffering from cardiovascular conditions, especially untreated hypertension, to abstain from using PY or PY-containing mushrooms. Furthermore, our results indicate that PY-induced ASC are generally well tolerated and integrated by healthy subjects.” (p. 155) |
| Barbosa et al. | 2005 | X |  |  |  |
| Moreno et al. | 2006 |  | X |  | “Subjects generally tolerated the procedure well. One subject experienced transient hypertension, which was not associated with psychic anxiety or somatic symptoms. Two subjects declined further participation after the first testing session due to discomfort with hospitalization. No other adverse reactions were observed.” (p. 1737) |
| Trichter et al. | 2009 | X |  |  |  |
| Griffiths et al. | 2011 |  | X |  | “There were no reports of bothersome or clinically significant persisting perception  phenomena sometimes attributed to hallucinogen use. Likewise, there were no reports of any non-study use of hallucinogens since study enrollment. All 18 volunteers appeared to continue to be psychiatrically healthy, high-functioning, productive members of society.” (p. 661) |
| Grob et al. | 2011 |  | X |  | “Safe physiological and psychological responses were documented during treatment sessions. There were no clinically significant adverse events with psilocybin.” (abstract) “We also observed no adverse psychological effects from the treatment. All subjects tolerated the treatment sessions well, with no indication of severe anxiety or a “bad trip.”“ (p. 77) |
| Frecska et al. | 2012 | X |  |  |  |
| Johnson et al. | 2014 |  |  | X | n = 8/10 reported at least one post-psilocybin headache:  “During the 42 psilocybin sessions (16 moderate, 26 high dose) conducted in the course of this study, no clinically significant adverse events requiring physician or pharmacologic intervention occurred. […] Visual Effects Questionnaire data showed no increase in the occurrence of clinically significant or bothersome visual effects, comparing intake and 6-month follow-up assessments. Of the ten participants assessed for headache, eight reported at least one post-psilocybin headache with a mean (SD; range) duration of 5.8 (2.4; 2.0–9.5) hours, onset at a mean of 6.2 (2.1; 3.5–11.5) hours after psilocybin administration and mean severity rating of mild, or 2.6 (1.3; 1–5) on a scale from 1=minimal, to 6=excruciating. Five reported use of over-the counter headache medication the evening following the session to alleviate symptoms. These were considered approved medications for use during the study, as there is no evidence to suggest they would affect smoking cessation outcomes.” (p. 7) |
| Bogen­schutz et al. | 2015 |  |  | X | n = 5 mild headaches, n = 1 insomnia:  “Five participants reported mild headaches which resolved within 24 hours following psilocybin administration […]. One participant reported insomnia on the night following a psilocybin session. No participant required medication or other intervention for blood pressure, anxiety, or other psychiatric symptoms. There was no report of illicit hallucinogen use by any participant during study participation.” (p. 294) |
| Osório et al. | 2015 |  | X |  | “AYA was well tolerated by all patients, suggesting that it can be safely administered to depressed patients. […] In the present study, the psychoactive effects of AYA were considered by participants as mild and short-lived, corroborating the nonsignificant effect of AYA on the BPRS-TD subscale. The nonsignificant increases in blood pressure replicate previous findings in human studies suggesting that AYA produces moderate cardiovascular effects.” (p. 18) |
| Schmid et al. | 2015 |  |  | X | List of Complaints 24-72 hours after LSD: Difficulty to concentrate (n=2), headache (n=2), exhaustion (n=3), Dry mouth (n=1) (Table S3)  “Adverse effects produced by LSD completely subsided within 72 hours. No  severe acute adverse effects were observed” (abstract)  “Adverse effects at 24–72 hours did not differ between LSD and placebo” (p. 548) |
| Schmid & Liechti^2^ | 2018 |  |  | X | n = 1 sleep disturbances, n = 1 vivid dreams:  “At the 1-month follow-up, one subject reported problems falling asleep and having more vivid dreams over 10 days after LSD administration. None of the participants reported any psychological problems or perceptual changes/disorders (e.g., flashbacks) up to 1 month after the LSD session.” (p. 541) |
| Carhart-Harris et al. | 2016 | X |  |  |  |
| Dolder et al. | 2016 |  |  | X | “There were no severe adverse events.” (p. 2641)  Subacute adverse effects 24-72h very low and no significant difference to placebo group. (Table 1) |
| Ross et al. | 2016 |  |  | X | “Regarding psychiatric AEs, no pharmacological interventions (e.g. benzodiazepines, anti-psychotics) were needed during dosing sessions, no participants abused or became addicted to psilocybin, there were no cases of prolonged psychosis or hallucinogen persisting perceptual disorder (HPPD), and no participants required psychiatric hospitalization. In terms of AEs attributable to psilocybin, the most common medical AEs were non-clinically significant elevations in BP and HR (76%), headaches/migraines (28%), and nausea (14%); the most common psychiatric AEs were transient anxiety (17%) and transient psychotic-like symptoms (7%: one case of transient paranoid ideation and one case of transient thought disorder). The medical AEs (non-clinically significant elevations in BP and HR, headaches, nausea), and psychiatric AEs (transient anxiety, transient near-psychotic symptoms) attributable to psilocybin are all known AEs of psilocybin, were transient, tolerable, and consistent with prior trials of psilocybin administration in normal volunteers (Griffiths et al., 2006, 2008, 2011), and patients with terminal cancer (Grob et al., 2011)” (p. 1173) |
| Sanches et al. | 2016 |  | X |  | “Ayahuasca was well tolerated. Blood pressure and heart rate were nonsignificantly increased (data not shown). Vomiting, reported by 47% of the volunteers, was the only adverse effect recorded. Ayahuasca effects on thought content and sensory perception were considered mild and short-lived, and no dysphoric effects were reported. Volunteers were calm and relaxed during acute drug effects, and considered the ayahuasca session as a pleasant experience.” (p. 79) |
| Soler et al. | 2016 | X |  |  |  |
| Sampe­dro et al. | 2017 | X |  |  |  |
| Carhart-Harris et al. | 2018 |  |  | X | n = 8 headaches lasting not more than 1-2 days:  “Treatment was generally well tolerated and there were no serious adverse events.” (p. 5)  “Consistent with our earlier report on the initial 12 patients from this trial (Carhart-Harris et al. 2016), transient anxiety lasting for minutes (n = 15) and headaches lasting no more than 1–2 days (n = 8) were the most common side effects. Five reported transient nausea but there were no cases of vomiting. Three reported transient paranoia within the duration of the acute drug experience but this was short-lived in every case. As with all our previous work with this compound, there were no reported cases of so-called flashbacks or persisting perceptual changes.” (p. 5) |
| Lyons & Carhart-Harris^1^ | 2018a |  | X |  | “No serious adverse events occurred as a result of the psilocybin administration.” (p. 3) |
|  |  |  |  |  |  |
| Lyons & Carhart-Harris^1^ | 2018b | X |  |  |  |
| Stroud et al.^1^ | 2018 | X |  |  |  |
| Haijen et al. | 2018 | X |  |  |  |
| Mans et al.^3^ | 2021 | X |  |  |  |
| Soler et al. | 2018 | X |  |  |  |
| Uthaug et al. | 2018 | X |  |  |  |
| Domínguez-Clavé et al. | 2019 |  | X |  | None of the participants reported any adverse psychopathological  effects related to the ayahuasca sessions (p.576). |
| Mason et al. | 2019 | X |  |  |  |
| Palhano-Fontes et al. | 2019 |  | X |  | “No serious adverse events were observed during or after dosing. Although 100% of the patients reported feeling safe, the ayahuasca session was not necessarily a pleasant experience. In fact, some patients reported the opposite, as the experience was accompanied by much psychological distress. Most patients reported nausea, and about 57% have vomited, although vomiting is traditionally not considered a side effect of ayahuasca, but rather part of a purging process (Tafur, 2017).” (p. 661) |
| Uthaug et al. | 2019 | X |  |  |  |
| Anderson et al. | 2020 |  |  | X | Post-Medication visit psilocybin-related adverse events in n=18 participants: Headache (n=8), Fatigue (n=2), Insomnia (n=2), anxiety exacerbation, methamphetamine relapse (n=1), post-traumatic stress flashback, tinnitus, nausea, panic and insomnia (n=1), nausea (n=1) (table 3, p. 6) |
| Barrett et al. | 2020 | X |  |  |  |
| Jiménez-Garrido et al. | 2020 |  | X |  | “General mid-term adverse effects were not observed in this study, although some secondary acute reactions were observed in some individual cases (e.g. anxiety) and will be reported in a separate paper.” |
| Murphy-Beiner & Soar | 2020 | X |  |  |  |
| Netzband et al. | 2020 | X |  |  |  |
| Uthaug et al. | 2020 |  |  | X | n = 2 tension, n = 1 insomnia  “On the 7-day follow-up, 27.3% (N= 3) of the sample reported adverse effects in the days following the session. One participant reported some affect and somatic tension in muscles, one participant reported difficulties sleeping (insomnia), and finally, one participant reported experiencing somatic tension in muscles, in the 7 days following their session.” (p. 777) |
| Zeifman et al. | 2020 | X |  |  | Study 1 |
| Zeifman et al. | 2020 | X |  |  | Study 2 |
| Davis et al. | 2021 |  |  | x | “There were no serious adverse events in this trial. […] Mild to moderate transient headache was reported […] after the subjective psilocybin effects had subsided after 14 of 48 sessions (29%).” (p. 486)  Other adverse events reported within two weeks after Sessions 1 and 2 that were rated by staff as possibly or probably related to psilocybin (Suppl. 2, etable 9)  Session 1: Headache 7 (29%), Physical Discomfort 1 (4%), Mild controllable muscle motion 1 (4%), Visual distortion 1 (4%)  Session 2: Headache 7 (29%), Visual distortion 2 (8%), Tenseness/soreness 2 (8%), Chest tightness 1 (4%), Vivid dreams 1 (4%), Altered body sensation 1 (4%) |
| Dos Santos et al. | 2021 |  |  | x | n = 2 headache  “Both volunteers who reported headache described that it started in the end of the experimental session, persisted until the following day, and resolved spontaneously (with no use of medication). Except for these sub-acute effects, all other effects occurred during the experimental […] were transient, and resolved spontaneously […] No serious adverse effects or dropouts were observed. “ (p. 545) |
| Mason et al. | 2021 | X |  |  |  |
|  |  |  |  |  |  |
| Schindler et al. | 2021 |  |  | X | In the next 24h developed: n = 2 tension/sore muscles, n = 5 general headache attack, n = 2 migraine attack (table 3, p.541)  “There were no serious or unexpected AEs in this study. […] In the 24 h after experimental sessions, both placebo and psilocybin administration were followed by tension/sore muscles, general headache attack, and migraine attack. There were no significant differences between placebo and psilocybin in the incidences of AEs  [...] During follow-up with subjects, there were no AEs warranting professional intervention.” (p.538) |
| Uthaug et al. | 2021 | X |  |  |  |
| Wießner et al. | 2021 | X |  |  |  |
| ^1^subsample of Carhart-Harris et al., 2018  ^2^same sample as Schmid et al., 2015  ^3^(Sub-)sample of Haijen et al., 2018 | | | | | |
